# A standardized, genome-guided MLST scheme for *Avibacterium paragallinarum*: enhanced epidemiological typing and validation against existing methods

**DOI:** 10.1128/jcm.01267-25

**Published:** 2026-02-10

**Authors:** Mostafa Ghanem, Alyssa Harris, Madhusudan Timilsina, Dhiraj Chundru, Michele Williams, Amro Hashish, Mohamed El-Gazzar

**Affiliations:** 1 Department of Veterinary Medicine, University of Maryland Department of Veterinary Medicine 212460 , College Park, Maryland, USA; 2 Department of Veterinary Diagnostic and Production Animal Medicine, College of Veterinary Medicine, Iowa State University 70724 , Ames, Iowa, USA; University of California, Davis, Davis, California, USA

**Keywords:** *Avibacterium paragallinarum*, infectious coryza, multilocus sequence typing (MLST), molecular epidemiology, outbreak investigation, PubMLST

## Abstract

*Avibacterium paragallinarum*, the causative agent of infectious coryza (IC), is an important respiratory pathogen of chickens with growing prevalence in commercial and backyard flocks. Current strain-typing methods, including classical serotyping and molecular approaches, such as ERIC-PCR or single-locus HPG2 typing, lack sufficient discriminatory power to investigate the epidemiology or population structure. To address this limitation, we developed a genome-guided multilocus sequence typing (MLST) scheme as a robust and portable tool for *A. paragallinarum* strain differentiation. Housekeeping genes were identified from 42 whole-genome sequences (WGS); 18 candidates were evaluated; and six were selected for the final MLST scheme. We used the scheme to differentiate 75 *A. paragallinarum* samples and compared its performance against classical HPG2-based typing, *ad hoc* core genome MLST (cgMLST), and the MLST scheme published by M. Guo, Y. Jin, H. Wang, X. Zhang, and Y. Wu (Vet Sci 11:208, 2024, https://doi.org/10.3390/vetsci11050208). The new MLST showed higher discriminatory power than HPG2 and outperformed Guo’s scheme with higher discriminatory power, particularly for characterizing the samples originating from North and South America. It also showed strong concordance with cgMLST clustering while being more practical for routine use. Overall, the six-locus MLST identified 31 sequence types across 75 samples, revealing epidemiologically meaningful clustering at regional and national scales and capturing temporal persistence of lineages. All allele definitions and sequence types have been deposited in PubMLST, ensuring standardized nomenclature and global accessibility. This scheme represents a reproducible, cost-effective, and globally applicable tool that enhances outbreak investigation, surveillance, and population studies of *A. paragallinarum*, bridging the gap between low-resolution traditional methods and resource-intensive whole-genome sequencing.

**IMPORTANCE:**

Infectious coryza (IC) caused by *Avibacterium paragallinarum* is a major respiratory disease of poultry that causes acute infection, reducing egg production and growth and resulting in significant economic losses in poultry production worldwide. Controlling IC depends on understanding how different strains spread and persist, yet current methods to differentiate strains are either unreliable or too costly for routine use. In this study, we developed a standardized multilocus sequence typing system that provides a simple, accurate, and globally accessible way to identify and compare strains of *A. paragallinarum*. This scheme identified important links between outbreaks at local and regional levels and showed that certain strains persisted over time. By making the scheme available through PubMLST, laboratories worldwide can use a common tool to track and investigate the pathogen. This accessible tool improves disease surveillance, supports outbreak investigations, and helps poultry producers and veterinarians respond more effectively to IC.

## INTRODUCTION

*Avibacterium paragallinarum*, a gram-negative bacterium of the *Pasteurellaceae* family, is the etiological agent of infectious coryza (IC), an acute and sometimes chronic upper respiratory disease in chickens (1). IC causes substantial economic losses worldwide primarily through reduced egg production in layers, growth retardation, and elevated condemnation rates in broilers. Clinically, the disease is characterized by facial swelling, nasal discharge, lacrimation, and reduced feed and water intake (2). The severity and course of infection are influenced by persistent infection, coinfection with other pathogens, and host factors, such as age and breed (1, 3, 4).

Historically, IC was considered a warm-weather disease largely restricted to the southeastern United States and California (1, 3, 5). In recent years, IC has been increasingly reported across U.S. poultry operations, including in states, such as Ohio, Iowa, Pennsylvania, Delaware, and Maryland (5–7). Several studies have also documented rising case numbers and more severe clinical presentations globally during the past decade (5, 8–13), highlighting the growing significance of this pathogen.

Given the increasing frequency of IC outbreaks, effective control strategies are essential. Control strategies rely on strict biosecurity, antimicrobials, and vaccination. Yet, antimicrobials frequently fail to eliminate *A. paragallinarum* from flocks, allowing recovered birds to shed the bacterium and serve as a source of reinfection, particularly in multi-age farms (14, 15). Although vaccination reduces clinical severity, outbreaks still occur in vaccinated flocks, suggesting reduced efficacy or vaccine failure (16, 17). These challenges highlight the need for continuous monitoring, reliable detection, and robust epidemiological surveillance tools to maintain *A. paragallinarum*-free flocks and limit disease spread.

Conventional PCR assays, such as those targeting the HPG2 region (18), and more recent probe-based real-time PCR assay targeting *recN* (7) provide sensitive and specific detection of *A. paragallinarum*. However, PCR cannot discriminate between strains, a crucial requirement for outbreak investigation and epidemiological tracing. *A. paragallinarum* is a fastidious bacterium requiring NAD and hemin for *in vitro* growth (2). It has been classified into Page’s three serogroups (A, B, C) (19) and Kume’s nine serovars (A-1, A-2, A-3, A-4, B-1, C-1, C-2, C-3, and C-4) (20). Variants of serogroup B (21, 22) and NAD-independent strains (23, 24) have also been reported, highlighting the diversity of this pathogen.

Serotyping is labor-intensive, requires bacterial isolation, suffers from low reproducibility across laboratories, and frequently results in non-typeable strains due to limited availability of antisera (25, 26). Notably, genotyping based on two regions of the HMTp210, a trimeric autotransporter adhesin, classified *A. paragallinarum* into 14 genogroups that correlate with the classical Page and Kume serotypes (27). However, *HMTp210*-based typing remains limited in predicting all serovars, and it does not provide precise epidemiological insight, such as strain-relatedness, geographical associations, and population structure.

Other molecular approaches, including DNA fingerprinting methods, such as restriction endonuclease analysis, ribotyping, and ERIC-PCR, have been employed for strain differentiation (10, 28–30). However, typing based on DNA fingerprinting is time-consuming, has low reproducibility, requires pure cultures, and has limited epidemiological utility (10, 31). These limitations underscore the need for robust, sequence-based typing approaches.

Multilocus sequence typing (MLST), which characterizes samples using sequence fragments from five to seven housekeeping genes (32), provides a universal, portable, and standardized framework for bacterial strain classification. By capturing allelic variation across multiple conserved loci, MLST enables high-resolution discrimination of strains and supports population structure analyses, thereby strengthening molecular surveillance and epidemiological investigations. In veterinary microbiology and diagnostic medicine, such enhanced resolution is essential for tracing transmission pathways, distinguishing outbreak-related strains, and improving the interpretation of clinical and field cases. Importantly, MLST can be performed directly on DNA extracted from upper respiratory tract clinical samples, including choanal cleft, nasal, and infraorbital sinus swabs (6, 33, 34), making it a practical tool for routine diagnostic workflows and outbreak response. Several MLST assays for fastidious poultry pathogens have been successfully developed following similar approaches, including Mycoplasma *synoviae* (35, 36), *Mycoplasma gallisepticum* (37), *Mycoplasma iowae* (38), and *Ornithobacterium rhinotracheale* (39). Although an MLST scheme for *A. paragallinarum* was recently published (40), its housekeeping loci were selected from broader *Pasteurellaceae* gene sets and the scheme grouped U.S. and Mexican samples together, indicating limited resolution for local and regional epidemiological investigations in North America.

Thus, the present study aims to develop an MLST scheme capable of accurately differentiating *A. paragallinarum* strains, elucidating epidemiology and population structure, and supporting investigation of infectious coryza outbreaks. This work is particularly relevant in the North American context, where existing typing approaches have shown limited resolution for tracking strain diversity and transmission dynamics (1, 9, 12, 41). Furthermore, we assess the performance of existing typing methods against the proposed MLST scheme and the core genome MLST, the current gold-standard method for bacterial strain typing.

## MATERIALS AND METHODS

### Collection of *A. paragallinarum* samples

A total of 75 *A. paragallinarum* samples were used in the study, consisting of 45 whole-genome sequence (WGS) data sets from the National Center for Biotechnology Information (NCBI) database in March 2023 and 30 additional samples, including 25 clinical samples (frozen isolates in glycerol, active culture plates, or PCR-positive samples in BHI broth) and five WGS data sets obtained from state diagnostic laboratories, including Salisbury Animal Health Laboratory (SAHL; Salisbury, Maryland); Iowa State Diagnostic Laboratory (Ames, Iowa), Lasher Laboratory (Georgetown, Delaware), Ohio Animal Diseases Diagnostic Laboratory (ADDL; Reynoldsburg, Ohio), and CEVA Animal Health (Lenexa, Kansas). The samples used in the study originated from diverse geographical locations, including 34 from the United States, 22 from China, four from Germany, three from Mexico, two each from Peru, Japan, England, Russia, and France, and one each from South Africa and Gabon. All samples were assigned a unique identification starting with AP, followed by a serial number, origin state or country, and the last two digits of the year of collection (Table S1). Genomic DNA was extracted from the 25 clinical samples, which were received as frozen isolates, active culture plates, or enrichment broth samples of PCR positive submissions derived from nasal swabs or choanal cleft swabs, using the QIAamp DNA Mini-Kit (Qiagen, Valencia, CA) according to the manufacturer’s protocol. Clinical samples were reconfirmed as *A. paragallinarum* using PCR following the protocol described by Chen et al. (18). The extracted DNA from the PCR-positive clinical samples was stored in the freezer at −20°C until further use.

### Identification of putative MLST loci

Putative MLST loci of *A. paragallinarum* were identified using an *ad hoc* core-genome MLST (cgMLST) scheme developed from 42 WGS available in NCBI GenBank as of April 2021 using Ridom SeqSphere+ (Version7.0.4.) (supplemental material). The genome of AP01_PE_FARPER_15 was selected as the reference, and the remaining 41 genomes were queried to screen for housekeeping genes. The housekeeping genes with the highest number of alleles were prioritized for further evaluation. Multiple sequence alignments of the genes were visually inspected to identify the loci on which primers can be designed at both ends to amplify the nested region containing nucleotide variations. All primers were designed to have an optimum annealing temperature of ∼60°C and used to amplify the candidate loci from the clinical samples. The specificity of the designed primers for each selected locus was evaluated using the NCBI primer-BLAST tool against the standard nr/nt database (https://www.ncbi.nlm.nih.gov/tools/primer-blast/). To determine the sensitivity of each primer set, genomic DNA of the AP10_DEL_20 was quantified using Qubit (Invitrogen, USA) and serially diluted from 10^7^ to 10^3^ genome copies/mL. The limit of detection (LoD) for the PCR primer set at each locus was defined as the lowest initial DNA concentration that produced a visible amplicon on agarose gel. Furthermore, the discriminatory power of each locus for strain differentiation was assessed using the Hunter-Gaston Diversity Index, a metric derived from Simpson’s Index of Diversity (42). Six loci with the highest discriminatory index (DI) and consistent amplification success were selected as the final loci for the MLST scheme.

### Amplification and sequencing of MLST loci

Targeted amplification and sequencing of the six MLST loci were carried out from 16 clinical samples received as PCR-positive aliquots derived from nasal swabs or choanal cleft swabs, where WGS was not feasible. Initially, PCR was performed in a 25 µL reaction containing 12.25 µL GoTaq Colorless Hot Start (Promega, USA), 1.25 µL primer mix (10 mM), 10.5 µL nuclease-free water, and 1 µL DNA template to amplify the selected MLST loci. PCR cycling parameters were optimized in our laboratory, including initial denaturation at 95°C for 2 min, followed by 35 cycles of 94°C for 30 s, 60°C for 30 s, and 72°C for 90 s, with a final extension at 72°C for 5 min. PCR amplicons were purified using the QIAquick PCR Purification Kit (Qiagen, USA) before sequencing. Sanger sequencing was performed on an ABI 3130/3130xl genetic analyzer following cycle sequencing with BigDye Terminator v3.1 chemistry (Applied Biosystems, USA) and subsequent cleanup using HighPrep DTR magnetic beads (MagBio Genomics, USA). The sequences were trimmed to avoid poor sequence quality associated with the ends of Sanger-sequenced amplicons.

### Whole-genome sequencing

From the clinical samples received as pure culture isolates, whole-genome sequencing libraries were prepared with Illumina DNA preparation kits and unique dual indexes (Illumina, USA). Sequencing was performed on the Illumina MiSeq platform (2 × 300 bp paired-end reads) at Ghanem Laboratory, University of Maryland. Raw reads were processed and assembled using the Bacterial and Viral Bioinformatics Resource Center (BV-BRC) pipeline (https://www.bv-brc.org/), with the assembly strategy set to “auto,” which performs a *de novo* assembly of Illumina reads using SPAdes as the default assembler (43, 44).

### MLST sequence analysis and sequence type (ST) assignment

MLST loci were identified and extracted from the whole-genome sequences (WGS), and allelic profiles were generated using the "Process WGS" function in Ridom SeqSphere+ (version 7.0.4). Reads from Sanger-sequenced clinical samples were also assembled and quality-checked using the same platform. Each unique sequence was assigned a new allele number, and each distinct allele combination across the six loci was assigned a unique sequence type (ST). Clonal complexes (CCs) were defined as groups of samples sharing at least four identical alleles with the ancestral ST of the cluster. MLST data analysis, including calculation of G+C content, number of alleles, number of polymorphic sites, percent DNA variability, and the discriminatory index (DI), was conducted using either the automated or manual tools available in SeqSphere+ (version 7.0.4). Minimum spanning tree (MST) and neighbor-joining dendrogram were generated based on the distance matrix from pairwise allelic profile comparison computed across the six MLST loci using the “pairwise, no missing values” option in Ridom SeqSphere+.

### HPG2 analysis

For comparison with the new MLST scheme, HPG2-based typing was performed using SeqSphere+ (version 7.0.4). The reference sequence of the HPG2 hypervariable region segment (444 nucleotides) was queried against 57 WGS samples. The HPG2 sequence from the clinical samples was generated by direct amplification and Sanger sequencing. The final minimum spanning tree (MST) and neighbor-joining dendrogram were generated for 68 samples, as an HPG2 gene could not be identified in seven WGS samples.

### cgMLST analysis for WGS samples

To further compare typing approaches, 57 WGS samples were typed using the *ad hoc* cgMLST scheme based on the 1,170 core genes (supplemental material). Each genome was screened for all targets using SeqSphere+ (Version 10.0.6), and MST and neighbor-joining dendrograms were generated for cluster visualization in the same software.

### Comparative evaluation with Guo et al. MLST scheme

To compare the performances across MLST schemes, we analyzed 57 *A. paragallinarum* WGS samples. SeqSphere (version 10.0.6) was used to process the genomes and assign sequence types based on the seven loci of Guo’s scheme and the six loci of our scheme. Minimum spanning trees were generated for each scheme, and their discriminatory power was evaluated for direct comparison.

### Data deposition in PubMLST

All allele sequences and sequence type (ST) profiles generated in this study have been deposited in the PubMLST database (https://pubmlst.org/organisms/avibacterium-paragallinarum). The database provides open access to allele definitions and ST assignments, enabling standardized nomenclature and facilitating a global comparison of the *A. paragallinarum* samples. This publicly available resource ensures that laboratories worldwide can adopt, validate, and expand the MLST scheme for epidemiological investigations of *A. paragallinarum*.

## RESULTS

### Selection and evaluation of MLST loci

**Fig 1:**
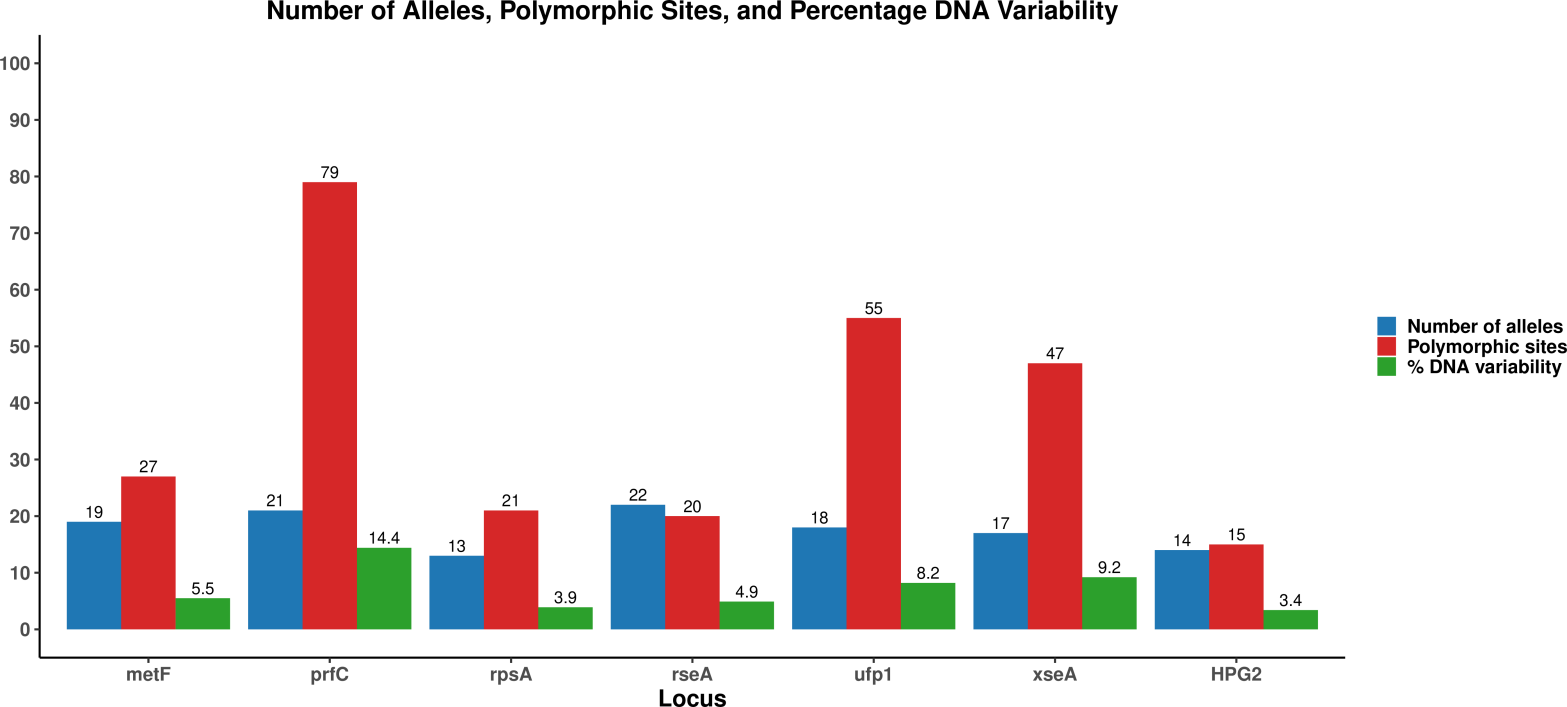
Number of alleles, polymorphic sites, and percentage of DNA variability in the six selected MLST loci and HPG2 sequence.

Based on the selection criteria, 18 housekeeping genes were initially identified as potential MLST loci from the 42 WGS of the *A. paragallinarum* samples. All 18 loci were successfully amplified from three clinical samples (Fig. S1), and their sequences were further analyzed. After evaluating combinations of seven loci in 42 samples, six loci were finally selected for the MLST scheme based on their discriminatory power: *metF*, *prfC*, *rpsA*, *rseA*, *ufp1*, and *xseA* (Fig. 1; Table 1). Primers designed for each of the loci were highly specific to *A. paragallinarum* with no cross-amplification from other species based on *in silico* analysis, and the limit of detection (LoD) for each primer set was 1.4 × 10^3^ genome copies per µL, indicating high analytical sensitivity. Based on these findings, the six selected loci were used for subsequent MLST typing of the complete 75-sample collection.

**TABLE 1:** Final six selected loci, concatenated sequence and their segment size, percent of G+C content, number of alleles, percent of DNA variability, polymorphic sites, and the Hunter-Gaston diversity index

	*metF*	*prfC*	*rpsA*	*rseA*	*ufp1*	*xseA*	Concatenated sequence
Segment size	495	549	534	408	669	513	3,168
% G+C content	45	50	36	43	47	49	45
Number of alleles	19	21	13	22	18	17	110
Polymorphic sites	27	79	21	20	55	47	249
% DNA variability	5.5	14.4	3.9	4.9	8.2	9.2	7.6
Diversity index (DI)	0.829	0.830	0.827	0.843	0.822	0.832	0.879
DI confidence interval (95% CI)	[0.772–0.887]	[0.777–0.890]	[0.772–0.882]	[0.786–0.900]	[0.762–0.881]	[0.774–0.890]	[0.823–0.934]

### MLST analysis

The MLST analysis of the 75 *A. paragallinarum* samples involved the sequence of the six selected loci (*metF*, *prfC*, *rpsA*, *rseA*, *ufp1*, and *xseA*), which resulted in the identification of 31 distinct sequence types (STs) (Table 2). Among the loci, *rseA* exhibited the highest allelic diversity with 22 alleles, followed by *prfC* (21), *metF* (19), *ufp1* (18), *xseA* (17), and *rpsA* (13). Among the six loci, *prfC* showed the highest nucleotide diversity (14.4%), followed by *xseA* (9.2%) and *ufp1* (8.2%), while *rpsA* exhibited the lowest variability at 3.9%. The discriminatory power of the individual loci based on the Hunter-Gaston Diversity Index (HGDI) ranged from 0.822 (*ufp1*) to 0.843 (*rseA*) (Fig. 1; Table 1).

**TABLE 2:** Thirty-one unique sequence types, their allelic profiles, and their frequency percentage out of the 75 *A. paragallinarum* samples used

Sequence type (ST)	*metF*	*prfC*	*rpsA*	*rseA*	*ufp1*	*xseA*	Frequency %
ST-1	1	1	1	1	1	1	1.3
ST-2	1	1	1	1	2	1	18.7
ST-3	1	1	2	2	2	2	2.7
ST-4	1	1	11	1	2	10	1.3
ST-5	1	16	1	1	2	1	1.3
ST-6	1	18	1	1	2	1	1.3
ST-7	2	2	3	3	3	3	29.3
ST-8	2	2	3	15	3	3	1.3
ST-9	3	3	4	4	4	4	1.3
ST-10	4	4	5	5	5	5	4.0
ST-11	5	5	6	6	6	6	4.0
ST-12	5	17	6	20	6	6	1.3
ST-13	5	20	6	6	6	6	1.3
ST-14	6	6	7	7	7	7	1.3
ST-15	7	7	8	8	8	8	4.0
ST-16	7	7	12	13	11	11	1.3
ST-17	7	8	8	8	4	9	1.3
ST-18	8	6	7	10	9	7	4.0
ST-19	8	6	7	11	9	7	1.3
ST-20	9	1	1	22	2	1	1.3
ST-21	10	10	10	12	10	5	1.3
ST-22	11	11	8	8	4	2	2.7
ST-23	12	2	3	3	3	3	1.3
ST-24	13	12	10	16	12	12	1.3
ST-25	13	21	9	9	18	17	1.3
ST-26	14	14	9	18	14	14	1.3
ST-27	15	15	10	19	15	15	1.3
ST-28	16	12	10	16	16	12	1.3
ST-29	17	13	9	17	13	13	1.3
ST-30	18	19	9	21	17	16	1.3
ST-31	19	9	13	14	14	15	1.3

The concatenated sequences of the six loci revealed an average nucleotide variability of 7.6%, compared to 3.4% of the single-locus HPG2 gene across the typed samples. The observed nucleotide variation reflects the combined polymorphisms across all six housekeeping genes. The G+C content of the MLST loci ranged from 36% (*rpsA*) to 50% (*prfC*). The calculated discriminatory index of the MLST scheme based on the concatenated sequences of the six loci was 0.879, which was higher than the DI observed for the single-locus HPG2 gene with the DI of 0.788 (Table 3), indicating that aggregating multiple independent genomic loci provides modestly higher epidemiological resolution than relying on a single hypervariable locus. To better understand the genetic relationships underlying these sequence types, cluster analysis was performed based on minimum spanning tree and neighbor-joining dendrogram.

**TABLE 3:** Number of whole genome- and target-sequenced *A. paragallinarum* samples, genotyping targets used, and discriminatory indices for the MLST scheme, HPG2, cgMLST, and Guo’s MLST schemes

Typing scheme	Number of samples	Skipped samples	Sequence types	Discriminatory index (DI)	Confidence interval (95% CI)
HPG2	68	0	12	0.788	[0.724–0.851]
Ghanem_MLST	75	0	31	0.879	[0.823–0.934]
cgMLST	57	0	47	0.988	[0.976–1.0]
Guo_MLST_WGS	57	3	11	0.781	[0.706–0.855]
Ghanem_MLST_WGS	57	0	17	0.807	[0.724–0.89]

### Cluster analysis of the *A. paragallinarum* samples

**Fig 2:**
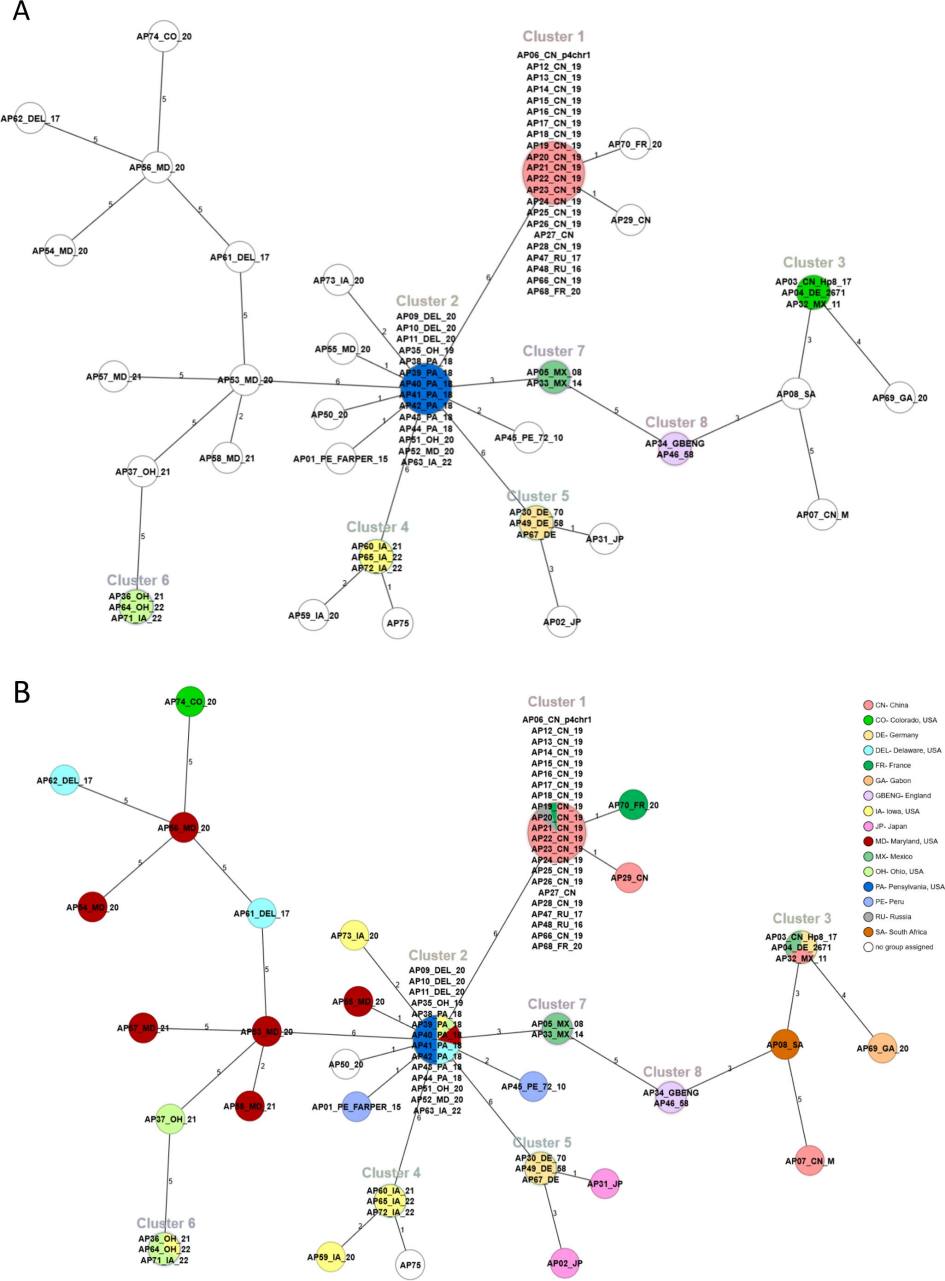
Minimum spanning tree of 75 *A. paragallinarum* samples based on the six-locus MLST generated using SeqSphere+. (**A**) Eight clusters identified from 75 *A. paragallinarum* samples as sequence types differing by no more than two alleles from the ancestral type; nodes are color-coded by matching cluster. Numbers on connecting lines indicate allele differences between the sequence types. (**B**) Minimum spanning tree of the 75 *A. paragallinarum* samples color-coded by geographic origin. Distances are based on pairwise allelic profile comparisons using the pairwise, no missing values option in SeqSphere+.

To investigate the population structure and the genetic diversity of *A. paragallinarum*, a minimum spanning tree (MST) was constructed using the allelic profiles of the 75 samples. This analysis revealed 31 distinct STs, each represented as a node in the MST. Sequence types sharing four or more alleles were grouped into clonal complexes, resulting in eight clonal clusters. The number of allelic differences between each ST is indicated on the connecting branches of the tree (Fig. 2A). The MST revealed both closely related and divergent lineages, supporting the high discriminatory capacity of the developed MLST scheme. The clustering pattern suggests a moderate level of genetic heterogeneity, consistent with the observed allelic and nucleotide diversity across the six selected loci.

**Fig 3:**
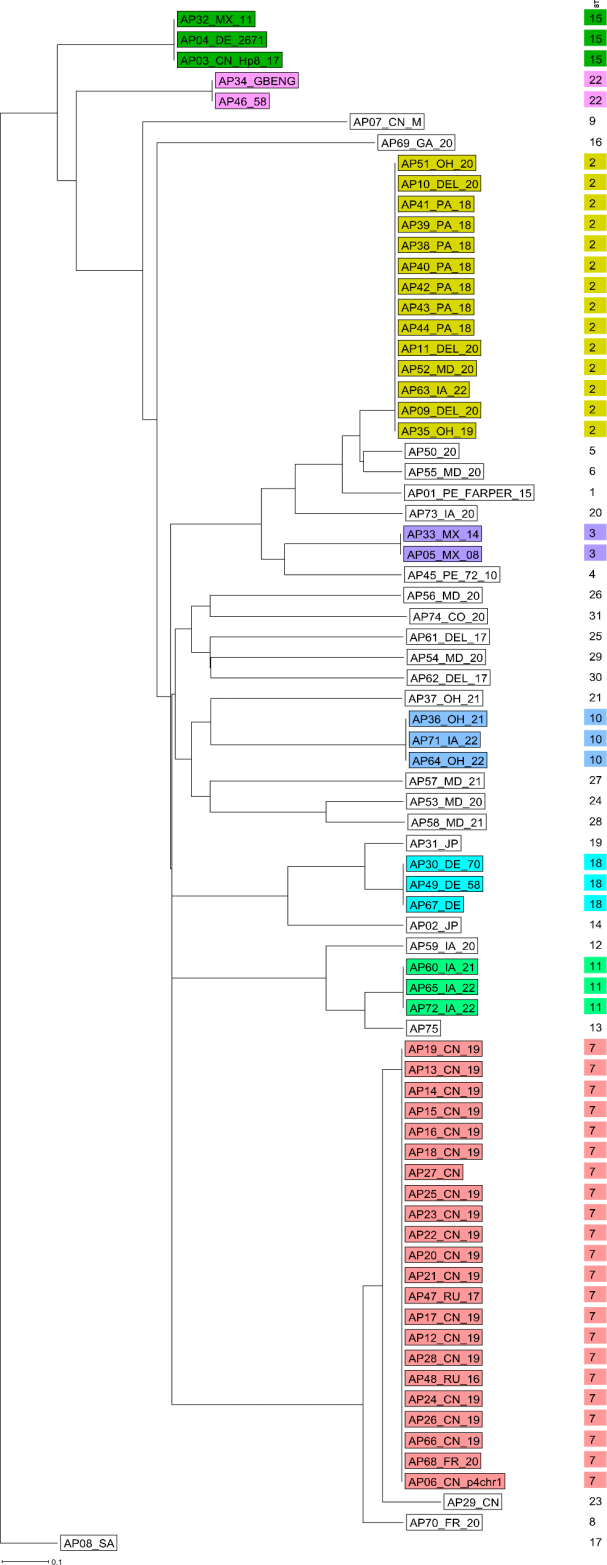
A six-locus MLST dendrogram displaying 75 samples, including Sanger-sequenced clinical samples. The distance matrix was built from all pairwise allelic profile comparisons of six MLST loci using the pairwise, no missing values option in SeqSphere+ software. The dendrogram is rooted by AP08_SA generated using the neighbor-joining method in SeqSphere+ software. Samples are color-coded according to their cluster, and ST of each sample is displayed to the right of the sample*.*

To further examine the geographical distribution of the samples, the MST was color-coded according to the sampling locations. While some clusters showed clear regional aggregation, others contained samples from multiple geographic origins, suggesting potential strain circulation across regions (Fig. 2B). In addition, a neighbor-joining (NJ) dendrogram generated from pairwise allelic profile comparisons and rooted on AP08_SA (SA-3) revealed clustering patterns that mirrored those in the MST, providing a consistent descriptive visualization of sample relationships inferred from the MLST scheme (Fig. 3). Because NJ was used without bootstrap support or evolutionary modeling, results are presented solely as descriptive clustering rather than phylogenetic inference. Furthermore, samples from backyard flocks in Maryland and Delaware showed little genetic relatedness, whereas samples from commercial layer and broiler flocks displayed close relatedness and clustered together (Fig. S2). While these analyses demonstrated the discriminatory ability of the MLST scheme, a comparative evaluation against existing typing methods was necessary to contextualize its performance.

### Comparative evaluation of the MLST scheme with HPG2 typing and cgMLST

**Fig 4:**
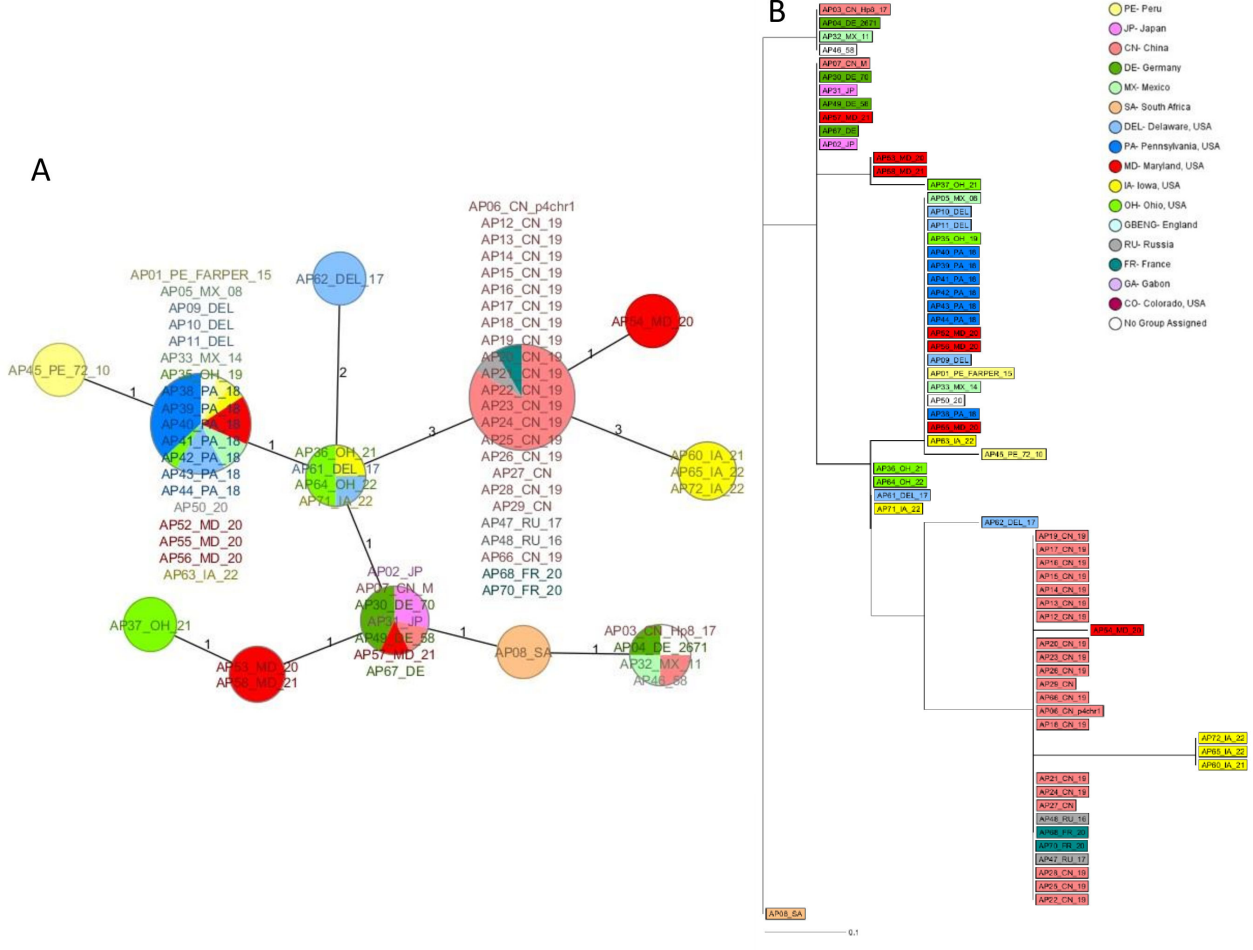
Clustering of the *A. paragallinarum* samples based on HPG2. (**A**) Minimum spanning tree generated for 68 samples based on the HPG2 sequence comparison. Distance based on nucleotide columns from 15 SNV positions with no missing values resulting in 12 sequence types. (**B**) MLST dendrogram generated for 68 samples based on the HPG2 sequence. Distance based on nucleotide columns from 15 SNV positions with no missing values and rooted to the AP08_SA sample. The dendrogram was generated using the neighbor-joining method in SeqSphere+ software*.* Samples are color-coded by geographic origin.

HPG2-based typing of 68 *A. paragallinarum* samples revealed comparatively lower discriminatory power, with a discriminatory index (DI) of 0.788, resulting in only 12 distinct sequence types. Several genetically and geographically diverse samples shared identical HPG2 profiles. For example, samples from Mexico, such as AP05_MX_08(ESV-135) and AP33_MX_14, and from Peru like AP01_PE_FARPER_15(FARPER-174) were grouped with U.S. samples like AP40_PA_18, AP52_MD_20, and AP63_IA_22 despite originating from distinct regions. Furthermore, the U.S. sample AP54_MD_20 clustered more closely with Chinese strains than with other U.S. samples (Fig. 4).

**Fig 5:**
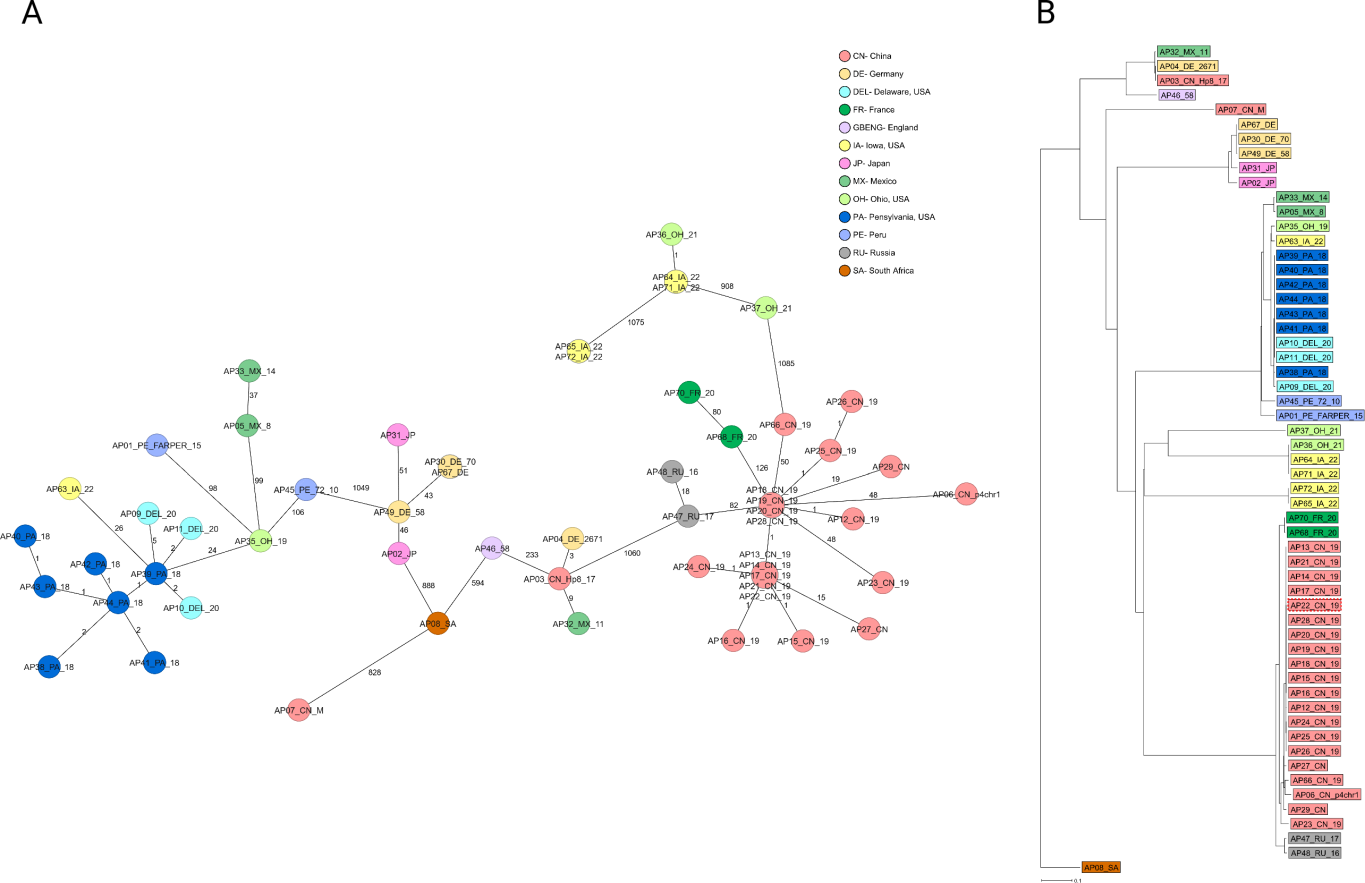
Clustering of the *A. paragallinarum* samples based on cgMLST. (**A**) Minimum spanning tree based on the allelic profiles of 1,170 core gene targets from 57 whole-genome samples using the SeqSphere+ software. (**B**) cgMLST dendrogram displaying 57 whole genome-sequenced *A. paragallinarum* samples. The distance matrix was built from all pairwise allelic profile comparisons of 1,170 cgMLST targets using the missing values as an own category option in SeqSphere+ software*.* Samples are color-coded by geographic origin, and dendrograms are rooted by the AP08_SA.

On the contrary, core genome MLST (cgMLST) analysis of 57 *A. paragallinarum* samples based on 1,170 conserved genes revealed substantial genetic diversity, resulting in 47 unique cgMLST types with a high discriminatory index (DI) of 0.988, significantly higher than that of HPG2 typing and MLST scheme. The cgMLST scheme showed an expected higher resolution than MLST, particularly in distinguishing closely related strains and resolving geographic clustering. Notably, the cgMLST dendrogram and MST revealed that the samples from China exhibited greater diversity compared to what was initially perceived as a single clonal complex using the MLST scheme (Fig. 5). Together, these results establish that the developed MLST scheme provides higher resolution than HPG2 while remaining substantially more accessible and practical than cgMLST for routine diagnostic and epidemiological use.

### Comparative evaluation with the existing MLST scheme of Guo et al. (2024)

**Fig 6:**
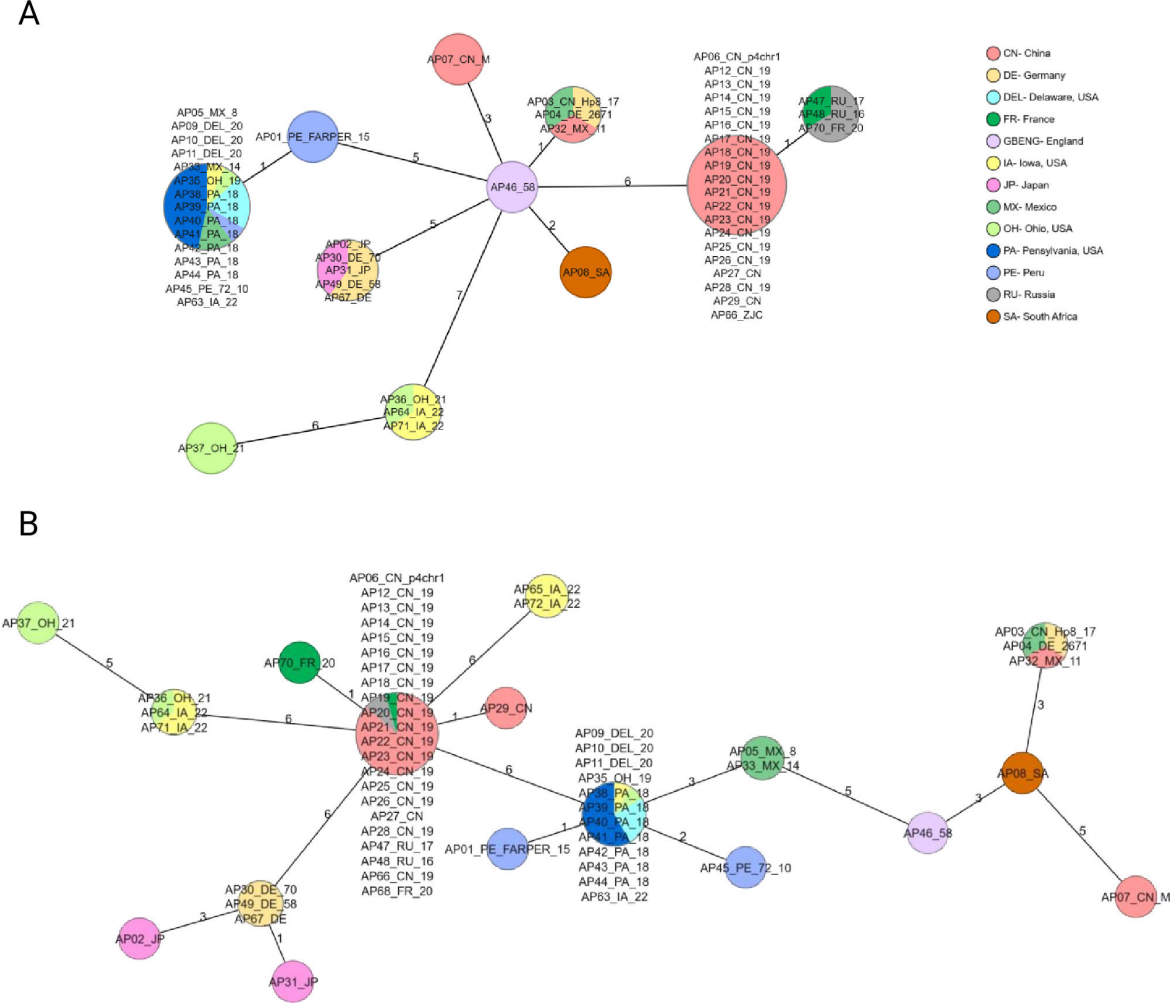
Minimum spanning tree of whole genome-sequenced samples based on two MLST profiles color-coded based on their geographic origin. (**A**) Minimum spanning tree of the WGS samples created using the seven-locus MLST scheme described by Guo et al. (40). (**B**) Minimum spanning tree of the WGS samples created using the MLST scheme developed in this study.

We analyzed 57 WGS to compare the performance of our MLST scheme with the existing seven-locus scheme of Guo et al. (40, 42). Using the same data set, our scheme differentiated all samples into 17 sequence types with higher discriminatory power (DI = 0.807) than Guo’s scheme, which assigned 54 samples to 11 STs and could not type three samples due to missing loci or quality-control failures (DI = 0.781). In the minimum spanning tree generated using Guo’s scheme, samples from Mexico and Peru clustered together with those from Pennsylvania, Delaware, Iowa, and Ohio, forming a single sequence type. In addition, three samples of German origin were also grouped together with two Japanese samples (Fig. 6A).

In contrast, our MLST scheme provided finer geographic resolution, differentiating U.S. samples at the state level and separating them from samples originating in Mexico, Peru, South Africa, Russia, and Germany. However, it also clustered samples from China with those from Russia and France, whereas Guo’s scheme resolved them, placing all Chinese samples, except for AP07_CN_M and AP03_CN_Hp8_17, in a separate group (Fig. 6B).

## DISCUSSION

Current strain differentiation methods for *A. paragallinarum*, including classical serotyping (19, 20) and molecular approaches like ERIC-PCR (30), have insufficient discriminatory power to investigate the epidemiology of outbreaks or population structure (10). Single-locus typing methods based on HPG2 have also shown limited resolution (1). We developed and evaluated a six-locus MLST scheme that provides higher discriminatory power and can be applied directly to DNA from clinical samples without prior culture, offering a practical and portable tool for infectious coryza diagnosis and epidemiological investigation. It is important to note that MLST functions as an epidemiological typing tool based on housekeeping gene variation and is not a marker of virulence or serotype. In contrast, targets, such as HPG2 and HMTp210, serve different biological and diagnostic roles, including species confirmation and antigen-associated serotyping, and do not provide phylogenetic resolution. Accordingly, our comparison with HPG2 was performed solely to evaluate relative epidemiological discriminatory power, as HPG2 sequencing has been widely used as a single-locus genotyping method in previous studies (10, 12, 33), and not to compare virulence or antigenic characteristics.

Compared to the single-locus HPG2 typing, the newly developed MLST demonstrated higher discriminatory power. HPG2-based typing frequently grouped genetically unrelated samples into the same sequence type and provided misleading geographical associations. For example, international samples, such as AP05_MX_08, AP33_MX_14, and AP01_PE_FARPER_15, shared identical HPG2 sequence types with U.S. samples AP40_PA_18, AP52_MD_20, AP35_OH_19, and AP63_IA_22. Similarly, a U.S. sample (AP54_MD_20) appeared genetically related to Chinese samples. In contrast, MLST consistently resolved these samples into distinct sequence types, indicating higher discriminatory power. The finding is consistent with earlier studies utilizing the HPG2-based strain typing, which identified only three sequence types in Mexico (12), five in the Netherlands (10), and two in Ethiopia (33), highlighting the potential limits of single-locus-based strain typing approaches. The greater discriminatory power observed with our MLST scheme reflects the combined allelic variation across six independent housekeeping loci. The concatenated loci exhibited 7.6% nucleotide variability across samples compared with 3.4% for HPG2. Multilocus approaches consistently outperform single-locus methods because they capture broader genomic diversity and reduce the risk of misleading phylogenetic inferences caused by recombination or locus-specific selection pressures. This principle has been well established in bacterial population genetics (32, 45, 46). In contrast, the HPG2 assay relies on a single genomic region with limited variability (3.4%), which restricts its epidemiological resolution.

Comparison with cgMLST confirmed the robustness of our six-locus MLST scheme. cgMLST, which interrogates 1,170 core genes, unsurprisingly provided even greater resolution, distinguishing, for example, a French sample (AP68_FR_20) from otherwise clonal Chinese samples with 126 allelic differences. Similar performance has been reported in other bacterial species (37, 47). However, cgMLST has practical limitations, including the requirement for pure isolates, high-quality whole-genome sequencing, and the logistical burden of maintaining large allele databases. These constraints are significant for *A. paragallinarum*, a fastidious organism that is difficult to culture. In contrast, our MLST offers an accessible, portable, and reproducible alternative that balances discriminatory power with practical feasibility.

*A. paragallinarum* is routinely detected from upper respiratory tract samples in diagnostic settings, including choanal cleft, nasal, and infraorbital sinus swabs using real-time PCR (7). This approach is widely adopted because *A. paragallinarum* is a fastidious organism, and practical constraints associated with bacterial isolation, such as transport time, temperature control, and the requirement for enrichment, often limit the feasibility of culture-based diagnosis. The same DNA extracted from PCR-positive diagnostic samples may potentially be suitable for downstream multilocus sequence typing, as demonstrated by a few of our clinical samples in this study and in previous studies (35–38). Because this method relies on targeted amplification of six housekeeping loci, it does not require culture isolation, although performance may decrease in samples with low bacterial load. This capability increases the practicality of MLST for routine diagnostics and outbreak investigations.

While this manuscript was in preparation, Guo et al. published an MLST scheme for *A. paragallinarum*, targeting seven loci selected from *Pasteurellaceae* gene sets (40). Although Guo’s scheme was effective in differentiating samples at broader geographic scales, it lacked the resolution to distinguish among North, Central, and South American samples and at the U. S. state level, thereby limiting its epidemiological utility, particularly within North and South America. These limitations likely stemmed from differences in sample selection for the scheme development, which were primarily of Chinese origin, as well as from differences in the locus selection strategy, which was initially based on the *Pasteurellaceae family conserved targets*. In contrast, our MLST scheme was developed using a genome-guided, *A. paragallinarum*-specific approach. Locus screening and selection were based on 42 whole-genome sequences of *A. paragallinarum*, which were further validated in the 33 additional samples, indicating that the selected loci are broadly conserved among the *A. paragallinarum* population. Overall, our six-locus MLST scheme developed in the current study provided greater discriminatory power than the seven-locus Guo et al. scheme while remaining slightly more economical and practical for diagnostic laboratories.

The epidemiological insights gained by our scheme illustrate its practical utility. MLST revealed a diverse population structure of *A. paragallinarum* globally and within the United States, with distinct clonal complexes often linked to geographic origin. Cluster 1 contained primarily Chinese samples with closely related but distinguishable strains from France and Russia. U.S. samples from Mid-Atlantic and Midwestern states (PA, MD, DE, OH, and IA) clustered in Cluster 2, suggesting that recent outbreaks may have originated from a closely related strain and potentially reflecting regional transmission dynamics. In contrast, multiple sequence types were observed among Maryland samples, likely reflecting circulation of diverse *A. paragallinarum* strains across different poultry operations. Importantly, samples from non-commercial flocks in Maryland and Delaware showed no genetic relatedness, consistent with the expected epidemiological patterns that backyard flocks have fewer transmission connections compared to commercial farms, where shared services (e.g., vaccine crews, feed trucks) may facilitate pathogen spread.

MLST also captured the temporal persistence of *A. paragallinarum* sequence types. For example, samples AP60_IA_21, AP65_IA_22, and AP72_IA_22 from Iowa, which were collected over two successive years, clustered together in Cluster 4. This indicates persistence and transmission of a single sequence type over time, consistent with the chronic carrier state of *A. paragallinarum* in multi-age layer facilities. Furthermore, our scheme correctly classified three public WGS samples submitted from different countries at different times into a single sequence type (AP03_CN_HP8_17, AP04_DE_2671, and AP32_MX_11), a result also confirmed by cgMLST, further validating the accuracy of our method. It is important to note that available metadata were limited and did not indicate whether samples originated from the same farm, neighboring farms, or different production companies, nor whether flocks included birds of multiple ages; therefore, clustering of identical or closely related sequence types may in part reflect unrecognized epidemiological linkage rather than independent transmission events.

We were unable to perform classical serotyping of our samples due to a lack of antisera. However, comparison with public GenBank data revealed no correlation between serotype and MLST sequence type. This is expected because MLST targets housekeeping genes that reflect evolutionary relationships, whereas serotyping reflects variations in surface antigens, such as HMTp210, which are more variable and subject to genetic exchange. As documented previously, samples from different regions may share identical serotypes despite limited genetic relatedness (48, 49).

In total, our scheme identified 31 distinct sequence types among 75 samples, providing an improved framework for studying the epidemiology, population structure, and transmission of *A. paragallinarum*. The scheme is non-ambiguous, portable, and globally accessible, requiring only amplification and sequencing of six loci that can be performed directly from clinical samples without isolation, making it suitable for routine diagnostics and outbreak investigations. All allele sequences and sequence types have been deposited into the PubMLST database, enabling standardized nomenclature and global data sharing.

Although MLST is not intended for vaccine selection, the sequence type information may help indicate whether circulating field samples are closely related to existing vaccine or reference strains, thereby supporting decisions related to updating regional or autogenous vaccines. However, serotyping methods, including HMTp210-based assays and classical HI tests, remain the appropriate tools for guiding vaccine formulation due to their antigenic relevance. In addition, MLST also has lower discriminatory power than cgMLST and provides only coarse phylogenetic resolution compared with cgMLST. As whole-genome sequencing becomes more accessible, a standardized cgMLST is expected to further improve strain typing. Until then, MLST offers a highly accurate and accessible alternative to complex, expensive, or technically challenging typing methods, meeting the immediate needs of *A. paragallinarum* epidemiology and outbreak investigation.

## Data Availability

All allele sequences and sequence type profiles generated in this study have been deposited in the PubMLST database for *Avibacterium paragallinarum* (https://pubmlst.org/organisms/avibacterium-paragallinarum). Additional data generated during the current study can be made available from the corresponding author upon request.

## SUPPLEMENTAL MATERIAL

Supplemental material jcm.01267-25-s0001.docx Figures S1 and S2 and supplemental data.

Table S1 jcm.01267-25-s0002.xlsx All 75 *A. paragallinarum* samples used for MLST scheme development and evaluation and their allelic profiles and other related information. Samples are color-coded according to their group in the main figures.

ASM does not own the copyrights to Supplemental Material that may be linked to, or accessed through, an article. The authors have granted ASM a non-exclusive, world-wide license to publish the Supplemental Material files. Please contact the corresponding author directly for reuse.
