# Supplementary material for: A standardized, genome-guided MLST scheme for Avibacterium paragallinarum: enhanced epidemiological typing and validation against existing methods
Source: J Clin Microbiol. 2026 Feb 10;64(3):e01267-25. doi: 10.1128/jcm.01267-25 (PMC12977546; doi:10.1128/jcm.01267-25)
Supplement: Supplemental material — Figures S1 and S2 and supplemental data. [file jcm.01267-25-s0001.docx]

**Supplementary Figures**


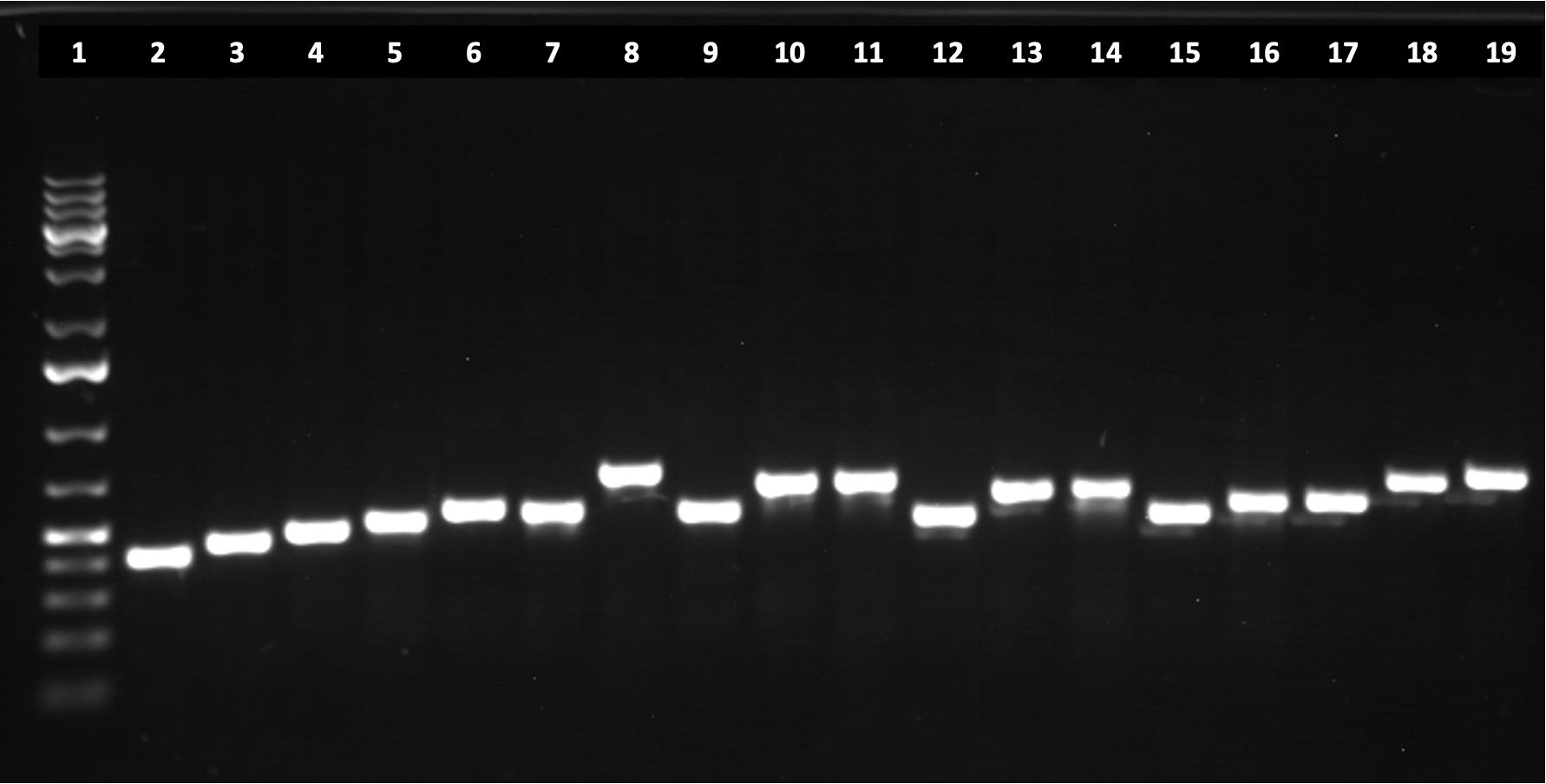


***Figure S1.*** *Agarose gel image of PCR amplicons of eighteen candidate loci that were successfully amplified with single bands using DNA from AP09_DEL_20. Lane 1 to 19 was loaded with GeneRuler 1 kb Plus DNA ladder, EIA51_00135, EIA51_00965, EIA51_01020, EIA51_02965, EIA51_03445, EIA51_04255, EIA51_06250, EIA51_06635, EIA51_06735, EIA51_07360, EIA51_07390, EIA51_07505, EIA51_08000, EIA51_08215, EIA51_08385, EIA51_08435, EIA51_10630, EIA51_10680*

***
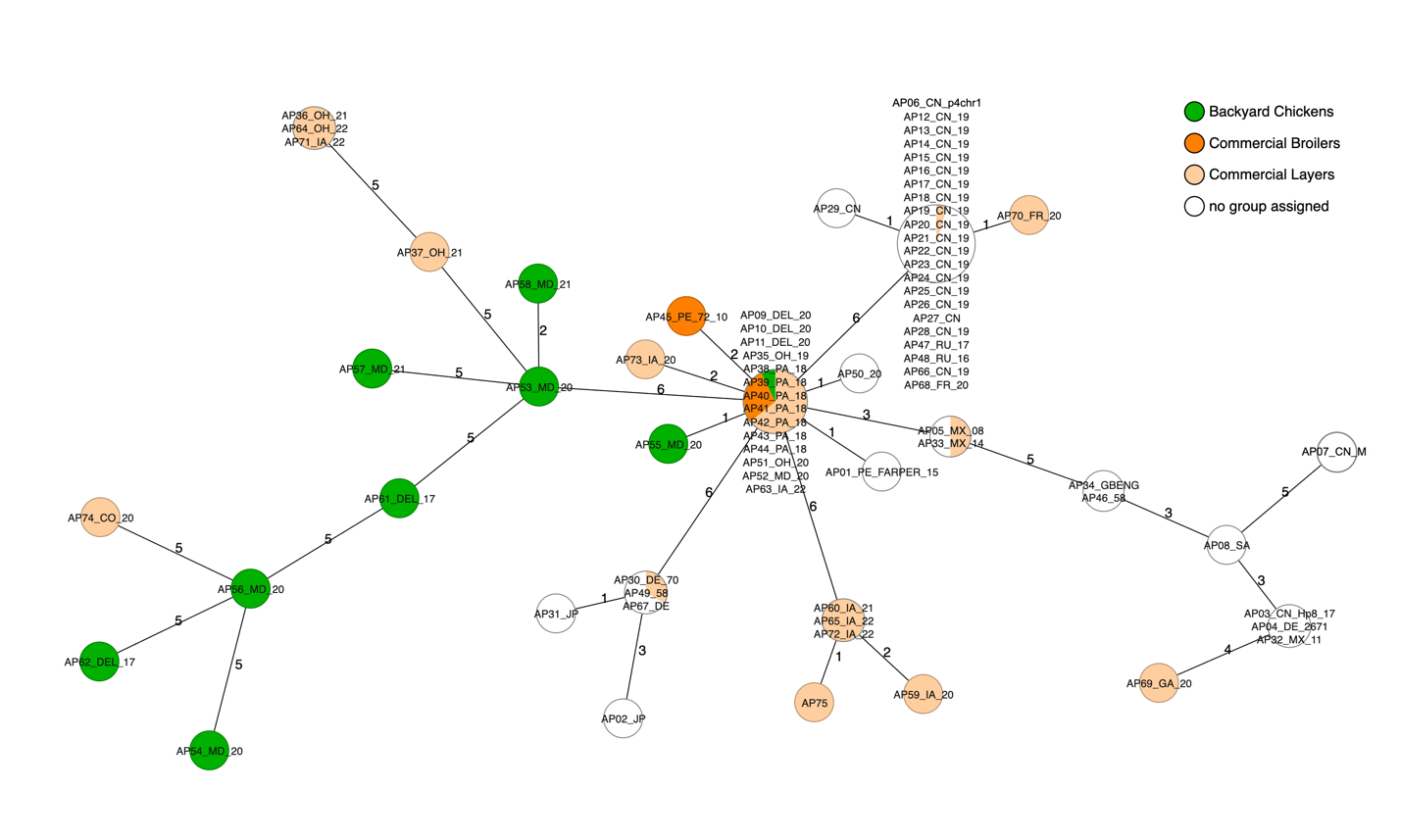
Figure S2.*** *Minimum spanning tree (MST) of 75 A. paragallinarum isolates based on six MLST loci. Nodes represent sequence types (ST) and are color-coded by production type. The tree was generated in SeqSphere+ using pairwise allelic profile distances from the six-locus MLST.*

## adhoc cgMLST Target Definer Results and list of core, accessory, and discarded genes.

Date: Apr 22, 2021, 3:42 PM

cgMLST Target Definer version: 1.5 (win)

Ridom SeqSphere+ version: 7.2.6

User: Mostafa Ghanem, University of Maryland

Server: 10.27.63.4 (SeqSphere+ Server on VDPAM-006219)

**Resulting Targets:**

1170 targets were defined for cgMLST (1097373 bases)

935 targets were used as Accessory targets (885978 bases)

148 targets were discarded.

**SETTINGS**

**Seed Genome:**

* NZ_CP034110.1 (06-JAN-2021), 2425949 bases, 2253 genes with CDS (Avibacterium paragallinarum strain FARPER-174 chromosome, complete genome)

**Penetration Query Genomes (41):**

* Fasta file 221.fasta, 2668385 bases, 135 contigs

* Fasta file 2671.fasta, 2337807 bases, 135 contigs

* Fasta file 72. fasta, 2453490 bases, 75 contigs

* Fasta file ADL-AP01.fasta, 2415542 bases

* Fasta file ADL-AP02.fasta, 2416187 bases

* Fasta file ADL-AP07.fasta, 2415993 bases

* Fasta file ADL-AP10.fasta, 2415552 bases

* Fasta file ADL-AP15.fasta, 2415950 bases

* Fasta file ADL-AP16.fasta, 2415855 bases

* Fasta file ADL-AP17.fasta, 2415699 bases

* Fasta file AP1-1N-1. fasta, 2592909 bases, 120 contigs

* Fasta file AP1-2N-1. fasta, 2593701 bases, 123 contigs

* Fasta file AP1-3N-1. fasta, 2593713 bases, 124 contigs

* Fasta file AP1-3S-1. fasta, 2593266 bases, 122 contigs

* Fasta file AP12-4N-1. fasta, 2592871 bases, 119 contigs

* Fasta file AP12-5S-1. fasta, 2593384 bases, 119 contigs

* Fasta file APX1-1N-2. fasta, 2592203 bases, 126 contigs

* Fasta file APX1-1S-1. fasta, 2593393 bases, 124 contigs

* Fasta file APX1-2N-1. fasta, 2592270 bases, 128 contigs

* Fasta file APX2-2N-2. fasta, 2594295 bases, 125 contigs

* Fasta file APX3-2S-2. fasta, 2592768 bases, 127 contigs

* Fasta file AVPG 221.fasta, 2655376 bases, 82 contigs

* Fasta file AVPG2015.fasta, 2532404 bases

* Fasta file CCUG 12835.fasta, 2460178 bases, 335 contigs

* Fasta file CL. fasta, 2410835 bases, 154 contigs

* Fasta file ESV-135.fasta, 2521134 bases

* Fasta file FARPER-174.fasta, 2425949 bases

* Fasta file GCF_015355095.1_ASM1535509v1_genomic.fna, 2593266 bases, 122 contigs

* Fasta file GCF_015355095.1_ASM1535509v1_genomic.fna.gz, 2593266 bases, 122 contigs

* Fasta file Hp8.fasta, 2357467 bases, 182 contigs

* Fasta file Modesto.fasta, 2499017 bases, 138 contigs

* Fasta file NCTC10926.fasta, 2790137 bases, 4 contigs

* Fasta file NCTC11296.fasta, 2860434 bases, 3 contigs

* Fasta file SA-3. fasta, 2390514 bases, 139 contigs

* Fasta file SCPM-O-B-8406.fasta, 2556353 bases, 67 contigs

* Fasta file SCPM-O-B-8407.fasta, 2554710 bases, 66 contigs

* Fasta file Y2S-2. fasta, 2563134 bases, 65 contigs

* Fasta file Z1N-1-2. fasta, 2592887 bases, 124 contigs

* Fasta file Z1N-2-1. fasta, 2593077 bases, 125 contigs

* Fasta file Z1N-2-2. fasta, 2591829 bases, 128 contigs

* Fasta file Z2S-1-2. fasta, 2591797 bases, 122 contigs

**Seed Genome Filters:**

* Minimum Length Filter (requires >=50 bases)

* Start Codon Filter (requires start codon at beginning of the gene)

* Stop Codon Filter (requires single stop codon at end of gene)

* Homologous Gene Filter (requires no multiple copies of gene with BLAST overlap>=100bp, identity>=90.0%)

* Gene Overlap Filter (requires no overlap with other genes >4 bases)

**Query Genome BLAST Search:**

* Requires BLAST hit with overlap=100%, identity>=90.0% in every query genome

* BLAST options: Word size=11, Mismatch penalty=-1, Match reward=1, Gap open costs=5, Gap extension costs=2

**Penetration Query Genomes Filters:**

* Stop Codon Percentage Filter (requires single stop codon at end of gene in >80% penetration query genomes)

BLAST version 2.2.12.

Citation: Altschul SF, Gish W, Miller W, Myers EW, Lipman DJ. 1990. Basic local alignment search tool. J Mol Biol 215:403-410.

DETAILS

Progress:

* **cgMLST target search start on Apr 22, 2021, 3:38 PM**

* 2253 targets found in seed genome NZ_CP034110.1 (06-JAN-2021), 2425949 bases, 2253 genes (Avibacterium paragallinarum strain FARPER-174 chromosome, complete genome)

* 1994 targets after filtering seed genome NZ_CP034110.1 (06-JAN-2021), 2425949 bases, 2253 genes (Avibacterium paragallinarum strain FARPER-174 chromosome, complete genome)

* 1519 targets after blasting against Fasta file 221.fasta, 2668385 bases, 135 contigs

* 1395 targets after blasting against Fasta file 2671.fasta, 2337807 bases, 135 contigs

* 1382 targets after blasting against Fasta file 72. fasta, 2453490 bases, 75 contigs

* 1377 targets after blasting against Fasta file ADL-AP01.fasta, 2415542 bases

* 1377 targets after blasting against Fasta file ADL-AP02.fasta, 2416187 bases

* 1377 targets after blasting against Fasta file ADL-AP07.fasta, 2415993 bases

* 1377 targets after blasting against Fasta file ADL-AP10.fasta, 2415552 bases

* 1377 targets after blasting against Fasta file ADL-AP15.fasta, 2415950 bases

* 1377 targets after blasting against Fasta file ADL-AP16.fasta, 2415855 bases

* 1377 targets after blasting against Fasta file ADL-AP17.fasta, 2415699 bases

* 1300 targets after blasting against Fasta file AP1-1N-1.fasta, 2592909 bases, 120 contigs

* 1300 targets after blasting against Fasta file AP1-2N-1.fasta, 2593701 bases, 123 contigs

* 1300 targets after blasting against Fasta file AP1-3N-1.fasta, 2593713 bases, 124 contigs

* 1300 targets after blasting against Fasta file AP1-3S-1.fasta, 2593266 bases, 122 contigs

* 1300 targets after blasting against Fasta file AP12-4N-1.fasta, 2592871 bases, 119 contigs

* 1300 targets after blasting against Fasta file AP12-5S-1.fasta, 2593384 bases, 119 contigs

* 1300 targets after blasting against Fasta file APX1-1N-2.fasta, 2592203 bases, 126 contigs

* 1300 targets after blasting against Fasta file APX1-1S-1.fasta, 2593393 bases, 124 contigs

* 1300 targets after blasting against Fasta file APX1-2N-1.fasta, 2592270 bases, 128 contigs

* 1300 targets after blasting against Fasta file APX2-2N-2.fasta, 2594295 bases, 125 contigs

* 1300 targets after blasting against Fasta file APX3-2S-2.fasta, 2592768 bases, 127 contigs

* 1300 targets after blasting against Fasta file AVPG 221.fasta, 2655376 bases, 82 contigs

* 1299 targets after blasting against Fasta file AVPG2015.fasta, 2532404 bases

* 1294 targets after blasting against Fasta file CCUG 12835.fasta, 2460178 bases, 335 contigs

* 1244 targets after blasting against Fasta file CL.fasta, 2410835 bases, 154 contigs

* 1244 targets after blasting against Fasta file ESV-135.fasta, 2521134 bases

* 1244 targets after blasting against Fasta file FARPER-174.fasta, 2425949 bases

* 1244 targets after blasting against Fasta file GCF_015355095.1_ASM1535509v1_genomic.fna, 2593266 bases, 122 contigs

* 1244 targets after blasting against Fasta file GCF_015355095.1_ASM1535509v1_genomic.fna.gz, 2593266 bases, 122 contigs

* 1243 targets after blasting against Fasta file Hp8.fasta, 2357467 bases, 182 contigs

* 1220 targets after blasting against Fasta file Modesto.fasta, 2499017 bases, 138 contigs

* 1198 targets after blasting against Fasta file NCTC10926.fasta, 2790137 bases, 4 contigs

* 1198 targets after blasting against Fasta file NCTC11296.fasta, 2860434 bases, 3 contigs

* 1181 targets after blasting against Fasta file SA-3.fasta, 2390514 bases, 139 contigs

* 1176 targets after blasting against Fasta file SCPM-O-B-8406.fasta, 2556353 bases, 67 contigs

* 1176 targets after blasting against Fasta file SCPM-O-B-8407.fasta, 2554710 bases, 66 contigs

* 1176 targets after blasting against Fasta file Y2S-2.fasta, 2563134 bases, 65 contigs

* 1176 targets after blasting against Fasta file Z1N-1-2.fasta, 2592887 bases, 124 contigs

* 1176 targets after blasting against Fasta file Z1N-2-1.fasta, 2593077 bases, 125 contigs

* 1176 targets after blasting against Fasta file Z1N-2-2.fasta, 2591829 bases, 128 contigs

* 1176 targets after blasting against Fasta file Z2S-1-2.fasta, 2591797 bases, 122 contigs

* cgMLST target search ended at Apr 22, 2021 3:42 PM

**cgMLST Genome Coverage:**

* 45.2% of Seed genome NZ_CP034110.1 (06-JAN-2021), 2425949 bases, 2253 genes with CDS (Avibacterium paragallinarum strain FARPER-174 chromosome, complete genome) bases covered by cgMLST targets

* 41.1% of Query genome Fasta file 221.fasta bases covered by cgMLST targets

* 46.9% of Query genome Fasta file 2671.fasta bases covered by cgMLST targets

* 44.7% of Query genome Fasta file 72.fasta bases covered by cgMLST targets

* 45.4% of Query genome Fasta file ADL-AP01.fasta bases covered by cgMLST targets

* 45.4% of Query genome Fasta file ADL-AP02.fasta bases covered by cgMLST targets

* 45.4% of Query genome Fasta file ADL-AP07.fasta bases covered by cgMLST targets

* 45.4% of Query genome Fasta file ADL-AP10.fasta bases covered by cgMLST targets

* 45.4% of Query genome Fasta file ADL-AP15.fasta bases covered by cgMLST targets

* 45.4% of Query genome Fasta file ADL-AP16.fasta bases covered by cgMLST targets

* 45.4% of Query genome Fasta file ADL-AP17.fasta bases covered by cgMLST targets

* 42.3% of Query genome Fasta file AP1-1N-1.fasta bases covered by cgMLST targets

* 42.3% of Query genome Fasta file AP1-2N-1.fasta bases covered by cgMLST targets

* 42.3% of Query genome Fasta file AP1-3N-1.fasta bases covered by cgMLST targets

* 42.3% of Query genome Fasta file AP1-3S-1.fasta bases covered by cgMLST targets

* 42.3% of Query genome Fasta file AP12-4N-1.fasta bases covered by cgMLST targets

* 42.3% of Query genome Fasta file AP12-5S-1.fasta bases covered by cgMLST targets

* 42.3% of Query genome Fasta file APX1-1N-2.fasta bases covered by cgMLST targets

* 42.3% of Query genome Fasta file APX1-1S-1.fasta bases covered by cgMLST targets

* 42.3% of Query genome Fasta file APX1-2N-1.fasta bases covered by cgMLST targets

* 42.3% of Query genome Fasta file APX2-2N-2.fasta bases covered by cgMLST targets

* 42.3% of Query genome Fasta file APX3-2S-2.fasta bases covered by cgMLST targets

* 41.3% of Query genome Fasta file AVPG 221.fasta bases covered by cgMLST targets

* 43.3% of Query genome Fasta file AVPG2015.fasta bases covered by cgMLST targets

* 44.6% of Query genome Fasta file CCUG 12835.fasta bases covered by cgMLST targets

* 45.5% of Query genome Fasta file CL.fasta bases covered by cgMLST targets

* 43.5% of Query genome Fasta file ESV-135.fasta bases covered by cgMLST targets

* 45.2% of Query genome Fasta file FARPER-174.fasta bases covered by cgMLST targets

* 42.3% of Query genome Fasta file GCF_015355095.1_ASM1535509v1_genomic.fna bases covered by cgMLST targets

* 42.3% of Query genome Fasta file GCF_015355095.1_ASM1535509v1_genomic.fna.gz bases covered by cgMLST targets

* 46.5% of Query genome Fasta file Hp8.fasta bases covered by cgMLST targets

* 43.9% of Query genome Fasta file Modesto.fasta bases covered by cgMLST targets

* 39.3% of Query genome Fasta file NCTC10926.fasta bases covered by cgMLST targets

* 38.4% of Query genome Fasta file NCTC11296.fasta bases covered by cgMLST targets

* 45.9% of Query genome Fasta file SA-3.fasta bases covered by cgMLST targets

* 42.9% of Query genome Fasta file SCPM-O-B-8406.fasta bases covered by cgMLST targets

* 43.0% of Query genome Fasta file SCPM-O-B-8407.fasta bases covered by cgMLST targets

* 42.8% of Query genome Fasta file Y2S-2.fasta bases covered by cgMLST targets

* 42.3% of Query genome Fasta file Z1N-1-2.fasta bases covered by cgMLST targets

* 42.3% of Query genome Fasta file Z1N-2-1.fasta bases covered by cgMLST targets

* 42.3% of Query genome Fasta file Z1N-2-2.fasta bases covered by cgMLST targets

* 42.3% of Query genome Fasta file Z2S-1-2.fasta bases covered by cgMLST targetstargets.

**TARGET DETAILS**

**Seed Genome Start Codon Filter: filtered out 10 targets (discarded)**

EIA51_RS01240, EIA51_RS01645, EIA51_RS02115, EIA51_RS05705, EIA51_RS08010,

EIA51_RS08660, EIA51_RS08790, EIA51_RS09900, EIA51_RS10130, EIA51_RS10890

**Seed Genome Stop Codon Filter: filtered out 79 targets (discarded)**

EIA51_RS00145, EIA51_RS00350, EIA51_RS00420, EIA51_RS00800, EIA51_RS01040,

EIA51_RS01385, EIA51_RS01415, EIA51_RS01600, EIA51_RS01880, EIA51_RS01950,

EIA51_RS02085, EIA51_RS02375, EIA51_RS02730, EIA51_RS02845, EIA51_RS02850,

EIA51_RS03015, EIA51_RS03025, EIA51_RS03040, EIA51_RS03085, EIA51_RS03355,

EIA51_RS03420, EIA51_RS03730, EIA51_RS03870, EIA51_RS03910, EIA51_RS04120,

EIA51_RS04285, EIA51_RS04345, EIA51_RS04430, EIA51_RS04815, EIA51_RS04965,

EIA51_RS04990, EIA51_RS05090, EIA51_RS05135, EIA51_RS05280, EIA51_RS05420,

EIA51_RS05505, EIA51_RS05540, EIA51_RS05570, EIA51_RS05740, EIA51_RS05860,

EIA51_RS05875, EIA51_RS05990, EIA51_RS06070, EIA51_RS06100, EIA51_RS06105,

EIA51_RS06140, EIA51_RS06215, EIA51_RS06240, EIA51_RS06255, EIA51_RS06305,

EIA51_RS06380, EIA51_RS06605, EIA51_RS06715, EIA51_RS06825, EIA51_RS06860,

EIA51_RS06895, EIA51_RS07120, EIA51_RS07315, EIA51_RS07485, EIA51_RS08025,

EIA51_RS08090, EIA51_RS09310, EIA51_RS09510, EIA51_RS09570, EIA51_RS09680,

EIA51_RS09710, EIA51_RS09765, EIA51_RS09770, EIA51_RS10135, EIA51_RS10390,

EIA51_RS10505, EIA51_RS10525, EIA51_RS10595, EIA51_RS10685, EIA51_RS11225,

EIA51_RS11400, EIA51_RS11760, EIA51_RS11815, EIA51_RS11820

**Seed Genome Homologous Gene Filter: filtered out 59 targets (discarded)**

EIA51_RS00020, EIA51_RS00060, EIA51_RS00065, EIA51_RS00770, EIA51_RS00900,

EIA51_RS01190, EIA51_RS01500, EIA51_RS01605, EIA51_RS02055, EIA51_RS02230,

EIA51_RS02235, EIA51_RS02270, EIA51_RS02320, EIA51_RS02325, EIA51_RS02330,

EIA51_RS02335, EIA51_RS02340, EIA51_RS02400, EIA51_RS02420, EIA51_RS02430,

EIA51_RS02450, EIA51_RS02535, EIA51_RS02675, EIA51_RS02700, EIA51_RS03150,

EIA51_RS04730, EIA51_RS07580, EIA51_RS07680, EIA51_RS07685, EIA51_RS07715,

EIA51_RS07740, EIA51_RS07805, EIA51_RS07840, EIA51_RS07845, EIA51_RS07850,

EIA51_RS07855, EIA51_RS07860, EIA51_RS07875, EIA51_RS07905, EIA51_RS07910,

EIA51_RS07960, EIA51_RS08855, EIA51_RS08945, EIA51_RS08970, EIA51_RS08990,

EIA51_RS09005, EIA51_RS10075, EIA51_RS10080, EIA51_RS10500, EIA51_RS10870,

EIA51_RS11255, EIA51_RS11260, EIA51_RS11580, EIA51_RS11630, EIA51_RS11770,

EIA51_RS11775, EIA51_RS11795, EIA51_RS11805, EIA51_RS11810

**Seed Genome Gene Overlap Filter: filtered out 111 targets (moved to Accessory)**

EIA51_RS00075, EIA51_RS00250, EIA51_RS00515, EIA51_RS00540, EIA51_RS00755,

EIA51_RS00790, EIA51_RS00825, EIA51_RS01000, EIA51_RS01065, EIA51_RS01070,

EIA51_RS01145, EIA51_RS01255, EIA51_RS01360, EIA51_RS01615, EIA51_RS01625,

EIA51_RS01635, EIA51_RS01655, EIA51_RS01825, EIA51_RS01890, EIA51_RS02105,

EIA51_RS02125, EIA51_RS02135, EIA51_RS02210, EIA51_RS02220, EIA51_RS02390,

EIA51_RS02550, EIA51_RS02585, EIA51_RS02650, EIA51_RS02680, EIA51_RS02980,

EIA51_RS03140, EIA51_RS03280, EIA51_RS03435, EIA51_RS03450, EIA51_RS03635,

EIA51_RS03845, EIA51_RS03890, EIA51_RS03995, EIA51_RS04140, EIA51_RS04300,

EIA51_RS04375, EIA51_RS04445, EIA51_RS04665, EIA51_RS04750, EIA51_RS04775,

EIA51_RS05040, EIA51_RS05250, EIA51_RS05385, EIA51_RS05400, EIA51_RS05455,

EIA51_RS05490, EIA51_RS05555, EIA51_RS05810, EIA51_RS05945, EIA51_RS06120,

EIA51_RS06160, EIA51_RS06185, EIA51_RS06325, EIA51_RS06420, EIA51_RS06480,

EIA51_RS06655, EIA51_RS06785, EIA51_RS06950, EIA51_RS06970, EIA51_RS07070,

EIA51_RS07130, EIA51_RS07215, EIA51_RS07275, EIA51_RS07325, EIA51_RS07745,

EIA51_RS07770, EIA51_RS07795, EIA51_RS07945, EIA51_RS08100, EIA51_RS08115,

EIA51_RS08130, EIA51_RS08190, EIA51_RS08225, EIA51_RS08280, EIA51_RS08375,

EIA51_RS08810, EIA51_RS08820, EIA51_RS08860, EIA51_RS08950, EIA51_RS09010,

EIA51_RS09150, EIA51_RS09265, EIA51_RS09275, EIA51_RS09395, EIA51_RS09580,

EIA51_RS09640, EIA51_RS09845, EIA51_RS10060, EIA51_RS10605, EIA51_RS10720,

EIA51_RS10945, EIA51_RS11040, EIA51_RS11055, EIA51_RS11215, EIA51_RS11320,

EIA51_RS11430, EIA51_RS11450, EIA51_RS11505, EIA51_RS11515, EIA51_RS11570,

EIA51_RS11600, EIA51_RS11615, EIA51_RS11720, EIA51_RS11745, EIA51_RS11755,

EIA51_RS11765

**Query Genome BLAST Search: filtered out 818 targets (moved to Accessory)**

EIA51_RS00010, EIA51_RS00015, EIA51_RS00025, EIA51_RS00030, EIA51_RS00035,

EIA51_RS00040, EIA51_RS00045, EIA51_RS00050, EIA51_RS00055, EIA51_RS00070,

EIA51_RS00105, EIA51_RS00110, EIA51_RS00185, EIA51_RS00190, EIA51_RS00195,

EIA51_RS00200, EIA51_RS00205, EIA51_RS00215, EIA51_RS00260, EIA51_RS00320,

EIA51_RS00325, EIA51_RS00335, EIA51_RS00340, EIA51_RS00345, EIA51_RS00355,

EIA51_RS00360, EIA51_RS00365, EIA51_RS00370, EIA51_RS00375, EIA51_RS00380,

EIA51_RS00385, EIA51_RS00390, EIA51_RS00395, EIA51_RS00400, EIA51_RS00405,

EIA51_RS00410, EIA51_RS00415, EIA51_RS00425, EIA51_RS00430, EIA51_RS00465,

EIA51_RS00495, EIA51_RS00505, EIA51_RS00520, EIA51_RS00535, EIA51_RS00565,

EIA51_RS00670, EIA51_RS00675, EIA51_RS00690, EIA51_RS00705, EIA51_RS00710,

EIA51_RS00715, EIA51_RS00720, EIA51_RS00725, EIA51_RS00730, EIA51_RS00735,

EIA51_RS00740, EIA51_RS00745, EIA51_RS00750, EIA51_RS00760, EIA51_RS00765,

EIA51_RS00780, EIA51_RS00785, EIA51_RS00795, EIA51_RS00840, EIA51_RS00850,

EIA51_RS00860, EIA51_RS00905, EIA51_RS00930, EIA51_RS00935, EIA51_RS00940,

EIA51_RS00945, EIA51_RS00990, EIA51_RS00995, EIA51_RS01005, EIA51_RS01025,

EIA51_RS01055, EIA51_RS01060, EIA51_RS01075, EIA51_RS01080, EIA51_RS01090,

EIA51_RS01125, EIA51_RS01130, EIA51_RS01135, EIA51_RS01205, EIA51_RS01225,

EIA51_RS01230, EIA51_RS01285, EIA51_RS01290, EIA51_RS01295, EIA51_RS01345,

EIA51_RS01355, EIA51_RS01370, EIA51_RS01390, EIA51_RS01405, EIA51_RS01435,

EIA51_RS01505, EIA51_RS01510, EIA51_RS01515, EIA51_RS01520, EIA51_RS01525,

EIA51_RS01530, EIA51_RS01535, EIA51_RS01540, EIA51_RS01545, EIA51_RS01550,

EIA51_RS01555, EIA51_RS01560, EIA51_RS01565, EIA51_RS01570, EIA51_RS01575,

EIA51_RS01580, EIA51_RS01585, EIA51_RS01590, EIA51_RS01595, EIA51_RS01610,

EIA51_RS01620, EIA51_RS01630, EIA51_RS01640, EIA51_RS01685, EIA51_RS01700,

EIA51_RS01750, EIA51_RS01765, EIA51_RS01770, EIA51_RS01790, EIA51_RS01820,

EIA51_RS01850, EIA51_RS01870, EIA51_RS01905, EIA51_RS01935, EIA51_RS01945,

EIA51_RS01995, EIA51_RS02000, EIA51_RS02015, EIA51_RS02060, EIA51_RS02065,

EIA51_RS02070, EIA51_RS02075, EIA51_RS02080, EIA51_RS02090, EIA51_RS02100,

EIA51_RS02110, EIA51_RS02120, EIA51_RS02130, EIA51_RS02140, EIA51_RS02145,

EIA51_RS02150, EIA51_RS02155, EIA51_RS02160, EIA51_RS02165, EIA51_RS02170,

EIA51_RS02175, EIA51_RS02180, EIA51_RS02185, EIA51_RS02190, EIA51_RS02195,

EIA51_RS02200, EIA51_RS02205, EIA51_RS02215, EIA51_RS02225, EIA51_RS02240,

EIA51_RS02245, EIA51_RS02250, EIA51_RS02255, EIA51_RS02260, EIA51_RS02265,

EIA51_RS02275, EIA51_RS02280, EIA51_RS02285, EIA51_RS02290, EIA51_RS02295,

EIA51_RS02305, EIA51_RS02310, EIA51_RS02315, EIA51_RS02345, EIA51_RS02350,

EIA51_RS02355, EIA51_RS02360, EIA51_RS02365, EIA51_RS02370, EIA51_RS02380,

EIA51_RS02395, EIA51_RS02405, EIA51_RS02410, EIA51_RS02415, EIA51_RS02425,

EIA51_RS02435, EIA51_RS02440, EIA51_RS02445, EIA51_RS02460, EIA51_RS02505,

EIA51_RS02510, EIA51_RS02515, EIA51_RS02540, EIA51_RS02545, EIA51_RS02555,

EIA51_RS02560, EIA51_RS02565, EIA51_RS02575, EIA51_RS02620, EIA51_RS02630,

EIA51_RS02635, EIA51_RS02640, EIA51_RS02645, EIA51_RS02655, EIA51_RS02660,

EIA51_RS02665, EIA51_RS02670, EIA51_RS02685, EIA51_RS02690, EIA51_RS02695,

EIA51_RS02720, EIA51_RS02725, EIA51_RS02860, EIA51_RS02870, EIA51_RS02875,

EIA51_RS02950, EIA51_RS03020, EIA51_RS03030, EIA51_RS03035, EIA51_RS03045,

EIA51_RS03050, EIA51_RS03055, EIA51_RS03060, EIA51_RS03065, EIA51_RS03070,

EIA51_RS03075, EIA51_RS03080, EIA51_RS03155, EIA51_RS03170, EIA51_RS03230,

EIA51_RS03250, EIA51_RS03260, EIA51_RS03310, EIA51_RS03315, EIA51_RS03320,

EIA51_RS03325, EIA51_RS03335, EIA51_RS03340, EIA51_RS03345, EIA51_RS03350,

EIA51_RS03360, EIA51_RS03365, EIA51_RS03370, EIA51_RS03390, EIA51_RS03500,

EIA51_RS03505, EIA51_RS03510, EIA51_RS03545, EIA51_RS03555, EIA51_RS03560,

EIA51_RS03565, EIA51_RS03570, EIA51_RS03575, EIA51_RS03585, EIA51_RS03645,

EIA51_RS03660, EIA51_RS03670, EIA51_RS03680, EIA51_RS03705, EIA51_RS03710,

EIA51_RS03715, EIA51_RS03735, EIA51_RS03795, EIA51_RS03800, EIA51_RS03805,

EIA51_RS03825, EIA51_RS03830, EIA51_RS03840, EIA51_RS03850, EIA51_RS03855,

EIA51_RS03860, EIA51_RS03865, EIA51_RS03960, EIA51_RS03975, EIA51_RS03980,

EIA51_RS04020, EIA51_RS04025, EIA51_RS04030, EIA51_RS04060, EIA51_RS04160,

EIA51_RS04190, EIA51_RS04240, EIA51_RS04275, EIA51_RS04280, EIA51_RS04290,

EIA51_RS04295, EIA51_RS04305, EIA51_RS04310, EIA51_RS04315, EIA51_RS04320,

EIA51_RS04325, EIA51_RS04330, EIA51_RS04335, EIA51_RS04340, EIA51_RS04385,

EIA51_RS04420, EIA51_RS04435, EIA51_RS04440, EIA51_RS04450, EIA51_RS04465,

EIA51_RS04470, EIA51_RS04475, EIA51_RS04480, EIA51_RS04485, EIA51_RS04490,

EIA51_RS04495, EIA51_RS04500, EIA51_RS04505, EIA51_RS04510, EIA51_RS04515,

EIA51_RS04520, EIA51_RS04525, EIA51_RS04530, EIA51_RS04575, EIA51_RS04580,

EIA51_RS04585, EIA51_RS04590, EIA51_RS04605, EIA51_RS04610, EIA51_RS04615,

EIA51_RS04620, EIA51_RS04625, EIA51_RS04645, EIA51_RS04650, EIA51_RS04655,

EIA51_RS04660, EIA51_RS04670, EIA51_RS04675, EIA51_RS04685, EIA51_RS04710,

EIA51_RS04715, EIA51_RS04725, EIA51_RS04735, EIA51_RS04755, EIA51_RS04780,

EIA51_RS04790, EIA51_RS04795, EIA51_RS04800, EIA51_RS04820, EIA51_RS04835,

EIA51_RS04840, EIA51_RS04845, EIA51_RS04880, EIA51_RS04885, EIA51_RS04890,

EIA51_RS04895, EIA51_RS04910, EIA51_RS04915, EIA51_RS05000, EIA51_RS05015,

EIA51_RS05020, EIA51_RS05025, EIA51_RS05050, EIA51_RS05055, EIA51_RS05080,

EIA51_RS05105, EIA51_RS05120, EIA51_RS05200, EIA51_RS05205, EIA51_RS05260,

EIA51_RS05265, EIA51_RS05270, EIA51_RS05315, EIA51_RS05335, EIA51_RS05390,

EIA51_RS05395, EIA51_RS05405, EIA51_RS05410, EIA51_RS05415, EIA51_RS05425,

EIA51_RS05440, EIA51_RS05475, EIA51_RS05480, EIA51_RS05495, EIA51_RS05500,

EIA51_RS05510, EIA51_RS05515, EIA51_RS05520, EIA51_RS05525, EIA51_RS05530,

EIA51_RS05535, EIA51_RS05560, EIA51_RS05565, EIA51_RS05600, EIA51_RS05605,

EIA51_RS05645, EIA51_RS05650, EIA51_RS05670, EIA51_RS05675, EIA51_RS05710,

EIA51_RS05725, EIA51_RS05730, EIA51_RS05735, EIA51_RS05745, EIA51_RS05780,

EIA51_RS05785, EIA51_RS05790, EIA51_RS05795, EIA51_RS05800, EIA51_RS05805,

EIA51_RS05815, EIA51_RS05835, EIA51_RS05905, EIA51_RS05930, EIA51_RS05970,

EIA51_RS05980, EIA51_RS05985, EIA51_RS06045, EIA51_RS06055, EIA51_RS06060,

EIA51_RS06065, EIA51_RS06085, EIA51_RS06090, EIA51_RS06095, EIA51_RS06110,

EIA51_RS06115, EIA51_RS06130, EIA51_RS06135, EIA51_RS06145, EIA51_RS06150,

EIA51_RS06155, EIA51_RS06170, EIA51_RS06175, EIA51_RS06220, EIA51_RS06245,

EIA51_RS06260, EIA51_RS06265, EIA51_RS06275, EIA51_RS06285, EIA51_RS06290,

EIA51_RS06310, EIA51_RS06320, EIA51_RS06330, EIA51_RS06360, EIA51_RS06390,

EIA51_RS06395, EIA51_RS06400, EIA51_RS06430, EIA51_RS06485, EIA51_RS06510,

EIA51_RS06525, EIA51_RS06600, EIA51_RS06615, EIA51_RS06620, EIA51_RS06630,

EIA51_RS06660, EIA51_RS06675, EIA51_RS06680, EIA51_RS06775, EIA51_RS06790,

EIA51_RS06795, EIA51_RS06830, EIA51_RS06835, EIA51_RS06845, EIA51_RS06865,

EIA51_RS06880, EIA51_RS06900, EIA51_RS06935, EIA51_RS06955, EIA51_RS06960,

EIA51_RS06990, EIA51_RS07005, EIA51_RS07010, EIA51_RS07015, EIA51_RS07020,

EIA51_RS07025, EIA51_RS07030, EIA51_RS07035, EIA51_RS07040, EIA51_RS07045,

EIA51_RS07050, EIA51_RS07055, EIA51_RS07065, EIA51_RS07075, EIA51_RS07150,

EIA51_RS07160, EIA51_RS07175, EIA51_RS07185, EIA51_RS07220, EIA51_RS07230,

EIA51_RS07235, EIA51_RS07240, EIA51_RS07245, EIA51_RS07250, EIA51_RS07255,

EIA51_RS07260, EIA51_RS07265, EIA51_RS07270, EIA51_RS07280, EIA51_RS07285,

EIA51_RS07290, EIA51_RS07385, EIA51_RS07395, EIA51_RS07400, EIA51_RS07410,

EIA51_RS07415, EIA51_RS07425, EIA51_RS07430, EIA51_RS07435, EIA51_RS07440,

EIA51_RS07450, EIA51_RS07470, EIA51_RS07475, EIA51_RS07480, EIA51_RS07490,

EIA51_RS07495, EIA51_RS07500, EIA51_RS07515, EIA51_RS07520, EIA51_RS07525,

EIA51_RS07535, EIA51_RS07540, EIA51_RS07545, EIA51_RS07550, EIA51_RS07555,

EIA51_RS07560, EIA51_RS07565, EIA51_RS07570, EIA51_RS07575, EIA51_RS07585,

EIA51_RS07590, EIA51_RS07595, EIA51_RS07600, EIA51_RS07605, EIA51_RS07610,

EIA51_RS07615, EIA51_RS07620, EIA51_RS07625, EIA51_RS07630, EIA51_RS07635,

EIA51_RS07640, EIA51_RS07645, EIA51_RS07650, EIA51_RS07655, EIA51_RS07660,

EIA51_RS07665, EIA51_RS07670, EIA51_RS07675, EIA51_RS07690, EIA51_RS07695,

EIA51_RS07700, EIA51_RS07720, EIA51_RS07725, EIA51_RS07730, EIA51_RS07735,

EIA51_RS07750, EIA51_RS07755, EIA51_RS07760, EIA51_RS07765, EIA51_RS07775,

EIA51_RS07780, EIA51_RS07785, EIA51_RS07790, EIA51_RS07800, EIA51_RS07810,

EIA51_RS07815, EIA51_RS07820, EIA51_RS07825, EIA51_RS07830, EIA51_RS07835,

EIA51_RS07865, EIA51_RS07870, EIA51_RS07880, EIA51_RS07890, EIA51_RS07895,

EIA51_RS07900, EIA51_RS07915, EIA51_RS07920, EIA51_RS07925, EIA51_RS07930,

EIA51_RS07935, EIA51_RS07940, EIA51_RS07950, EIA51_RS07955, EIA51_RS07965,

EIA51_RS07970, EIA51_RS07975, EIA51_RS08015, EIA51_RS08030, EIA51_RS08035,

EIA51_RS08040, EIA51_RS08045, EIA51_RS08050, EIA51_RS08055, EIA51_RS08080,

EIA51_RS08120, EIA51_RS08125, EIA51_RS08135, EIA51_RS08145, EIA51_RS08150,

EIA51_RS08160, EIA51_RS08175, EIA51_RS08180, EIA51_RS08220, EIA51_RS08245,

EIA51_RS08265, EIA51_RS08270, EIA51_RS08275, EIA51_RS08310, EIA51_RS08315,

EIA51_RS08325, EIA51_RS08335, EIA51_RS08395, EIA51_RS08400, EIA51_RS08405,

EIA51_RS08420, EIA51_RS08425, EIA51_RS08480, EIA51_RS08510, EIA51_RS08580,

EIA51_RS08635, EIA51_RS08640, EIA51_RS08745, EIA51_RS08785, EIA51_RS08795,

EIA51_RS08800, EIA51_RS08805, EIA51_RS08815, EIA51_RS08825, EIA51_RS08830,

EIA51_RS08835, EIA51_RS08840, EIA51_RS08845, EIA51_RS08850, EIA51_RS08890,

EIA51_RS08895, EIA51_RS08900, EIA51_RS08905, EIA51_RS08910, EIA51_RS08915,

EIA51_RS08920, EIA51_RS08935, EIA51_RS08940, EIA51_RS08955, EIA51_RS08960,

EIA51_RS08965, EIA51_RS08975, EIA51_RS08985, EIA51_RS08995, EIA51_RS09000,

EIA51_RS09015, EIA51_RS09030, EIA51_RS09110, EIA51_RS09170, EIA51_RS09175,

EIA51_RS09205, EIA51_RS09215, EIA51_RS09220, EIA51_RS09235, EIA51_RS09240,

EIA51_RS09250, EIA51_RS09255, EIA51_RS09260, EIA51_RS09280, EIA51_RS09305,

EIA51_RS09315, EIA51_RS09320, EIA51_RS09370, EIA51_RS09375, EIA51_RS09380,

EIA51_RS09385, EIA51_RS09390, EIA51_RS09400, EIA51_RS09405, EIA51_RS09410,

EIA51_RS09440, EIA51_RS09470, EIA51_RS09500, EIA51_RS09515, EIA51_RS09540,

EIA51_RS09545, EIA51_RS09555, EIA51_RS09635, EIA51_RS09645, EIA51_RS09650,

EIA51_RS09655, EIA51_RS09660, EIA51_RS09665, EIA51_RS09685, EIA51_RS09690,

EIA51_RS09700, EIA51_RS09705, EIA51_RS09715, EIA51_RS09725, EIA51_RS09745,

EIA51_RS09775, EIA51_RS09785, EIA51_RS09795, EIA51_RS09815, EIA51_RS09910,

EIA51_RS09915, EIA51_RS09920, EIA51_RS09925, EIA51_RS09940, EIA51_RS09945,

EIA51_RS09950, EIA51_RS09955, EIA51_RS10015, EIA51_RS10095, EIA51_RS10100,

EIA51_RS10105, EIA51_RS10110, EIA51_RS10115, EIA51_RS10185, EIA51_RS10205,

EIA51_RS10225, EIA51_RS10230, EIA51_RS10235, EIA51_RS10250, EIA51_RS10265,

EIA51_RS10280, EIA51_RS10285, EIA51_RS10305, EIA51_RS10365, EIA51_RS10370,

EIA51_RS10375, EIA51_RS10395, EIA51_RS10415, EIA51_RS10425, EIA51_RS10470,

EIA51_RS10475, EIA51_RS10480, EIA51_RS10485, EIA51_RS10490, EIA51_RS10495,

EIA51_RS10510, EIA51_RS10535, EIA51_RS10550, EIA51_RS10555, EIA51_RS10575,

EIA51_RS10600, EIA51_RS10620, EIA51_RS10635, EIA51_RS10650, EIA51_RS10655,

EIA51_RS10660, EIA51_RS10715, EIA51_RS10745, EIA51_RS10810, EIA51_RS10845,

EIA51_RS10850, EIA51_RS10895, EIA51_RS10900, EIA51_RS10905, EIA51_RS10955,

EIA51_RS10960, EIA51_RS10965, EIA51_RS10980, EIA51_RS10990, EIA51_RS11105,

EIA51_RS11140, EIA51_RS11145, EIA51_RS11150, EIA51_RS11200, EIA51_RS11300,

EIA51_RS11305, EIA51_RS11310, EIA51_RS11315, EIA51_RS11325, EIA51_RS11330,

EIA51_RS11335, EIA51_RS11340, EIA51_RS11345, EIA51_RS11350, EIA51_RS11355,

EIA51_RS11360, EIA51_RS11365, EIA51_RS11370, EIA51_RS11375, EIA51_RS11380,

EIA51_RS11385, EIA51_RS11390, EIA51_RS11395, EIA51_RS11405, EIA51_RS11410,

EIA51_RS11415, EIA51_RS11420, EIA51_RS11425, EIA51_RS11435, EIA51_RS11440,

EIA51_RS11445, EIA51_RS11455, EIA51_RS11460, EIA51_RS11465, EIA51_RS11470,

EIA51_RS11475, EIA51_RS11480, EIA51_RS11485, EIA51_RS11490, EIA51_RS11495,

EIA51_RS11500, EIA51_RS11510, EIA51_RS11520, EIA51_RS11525, EIA51_RS11530,

EIA51_RS11535, EIA51_RS11540, EIA51_RS11550, EIA51_RS11555, EIA51_RS11560,

EIA51_RS11565, EIA51_RS11585, EIA51_RS11590, EIA51_RS11595, EIA51_RS11605,

EIA51_RS11610, EIA51_RS11620, EIA51_RS11625, EIA51_RS11660, EIA51_RS11665,

EIA51_RS11680, EIA51_RS11685, EIA51_RS11700, EIA51_RS11705, EIA51_RS11725,

EIA51_RS11740, EIA51_RS11780, EIA51_RS11785

**Penetration Query Genomes Stop Codon Percentage Filter: filtered out 6 targets (moved to Accessory)**

EIA51_RS01235, EIA51_RS03675, EIA51_RS07370, EIA51_RS08195, EIA51_RS11100,

EIA51_RS11750

Messages for NZ_CP034110.1 (06-JAN-2021), 2425949 bases, 2253 genes with CDS (Avibacterium paragallinarum strain FARPER-174 chromosome, complete genome):

* Skipped EIA51_RS00005 because location is not contiguous.
